# Steroidomics in Men with Schizophrenia

**DOI:** 10.3390/ijms25168729

**Published:** 2024-08-10

**Authors:** Martin Hill, Marta Velíková, Tereza Hovorková, Josef Bulant, Katarína Janšáková, Karel Valeš

**Affiliations:** 1 Department of Steroids and Proteofactors, Institute of Endocrinology, Narodni 139/8, 110 00 Prague, Czech Republic; mvelikova@endo.cz (M.V.); tera.hovorkova@seznam.cz (T.H.); jbulant@endo.cz (J.B.); 2 Institute of Physiology, Faculty of Medicine, Comenius University in Bratislava, 811 08 Bratislava, Slovakia; jansakova.katarina@gmail.com; 3 National Institute of Mental Health, Topolova 748, 250 67 Klecany, Czech Republic; karel.vales@nudz.cz; 4 Department of Psychiatry and Medical Psychology, Third Faculty of Medicine, Charles University, 100 00 Prague, Czech Republic

**Keywords:** steroidomics, schizophrenia, GC-MS/MS, multivariate statistics, differential diagnostics

## Abstract

Schizophrenia is associated with numerous abnormalities, including imbalances in all hormonal axes, among which steroids play a major role. Steroidomic studies therefore represent a promising tool for early diagnosis and appropriate treatment of schizophrenia. A total of 51 adult male schizophrenics aged 27 (22, 34) years (shown as median with quartiles) and 16 healthy controls (HCs) aged 28 (25, 32) years were enrolled into this study. Our results showed the effective differentiation of men with schizophrenia from controls based on steroidomic profiles. We also found an altered metabolic pathway from pregnenolone and its sulfate (PREG/S) to cortisol in schizophrenics with several metabolic bottlenecks such as lower PREG levels due to increased PREG sulfation and/or suppressed PREGS desulfation and attenuated conversion of 17-hydroxy-PREG to 17-hydroxy-progesterone, as well as the results suggestive of suppressed CYP11B1 activity. In contrast, steroid molar ratios suggested two counterregulatory steps involving increased conversion of PREG/S to 17-hydroxy-PREG/S and decreased conversion of cortisol to cortisone, which may maintain unchanged basal cortisol levels but may not ensure a sufficient cortisol response to stress. Our data also indicated a trend to higher 7α-, 7β-, and 16α-hydroxylation that may counteract the autoimmune complications and proinflammatory processes accompanying schizophrenia. Finally, a possible suppression of HSD17B3 activity was suggested, resulting in decreased circulating testosterone levels with increased androstenedione levels.

## 1. Introduction

Schizophrenia is a persistent psychiatric disease with a prevalence of about 7‰ worldwide. It is characterized by positive (or psychotic) symptoms such as hallucinations, delusions, and disorganized speech, and negative symptoms including decreased motivation, diminished expressiveness, the blunted affect, apathy, attentional deficit, poverty of thought and speech, and social withdrawal (see reviews [1,2,3,4,5]).

Although the dopamine model of schizophrenia is incomplete on its own, it is still considered the most likely explanation for the cause of schizophrenia. This model is based on excessive activity of the mesolimbic dopaminergic pathway which is responsible for the development of the positive symptoms of schizophrenia [4]. Progesterone (P), may participate in the modulation of the dopamine system, either directly or via the GABAergic system as the increased brain levels of allopregnanolone (ALLO) dampen the dopamine release in dopaminergic regions [6]. Schizophrenia is linked to weakened glutamatergic activity [7] but some patients may display glutamate-mediated excitotoxicity in hippocampal and cortical regions [6].

Hyperactivity of the serotonergic system in schizophrenia may also explain some aspects of the disease in patients with positive symptoms of schizophrenia [7]. On the contrary, patients with impaired cognitive functions and negative symptoms have low serotonergic activity [8]. P and ALLO are 5-HT_3_ receptor antagonists, and their low levels may elicit excessive serotonergic activity [7]. The effects of the serotonergic system and the hypothalamic-pituitary-adrenal (HPA) axis may overlap [1]. Schizophrenia is associated with imbalances in all hormonal axes, among which steroids play a major role. A number of studies have been published on changes in steroid levels in the context of schizophrenia, including ideas about the mechanisms of these relationships, but these studies are often conflicting [3,4,6,9,10,11,12].

Neuroactive steroids (NASs) originating in the CNS (neurosteroids) as well as those of adrenal, gonadal, and peripheral origin, operating in both the central (CNS) and peripheral nervous systems (PNS), can suppress excitotoxicity, inflammatory processes, and oxidative stress and in turn counteract neurodegeneration. In addition, NASs accelerate neurogenesis and myelination [6]. Inflammation and an imbalance between the antioxidant defense system and the production of reactive oxygen species (ROS) are closely related to chronic stress and may contribute to the development of schizophrenia. There is also a relationship between schizophrenia and the major histocompatibility complex playing a vital role in adaptive and innate immune functions [1].

Pregnenolone (PREG), dehydroepiandrosterone (DHEA), and their sulfates may be involved in the pathophysiology of schizophrenia [13]. These steroids cross the blood-brain barrier, and their adrenal production and/or therapeutic supplementation may affect their concentrations in the CNS [12,14]. Elevated levels of PREG sulfate (PREGS), along with increased cortisol, reflect increased activity of the HPA axis in patients with schizophrenia in the first episode [15]. PREG is a pro-cognitive and neuroprotective against glutamate-induced neurotoxicity [6]. PREGS and DHEA sulfate (DHEAS) positively modulate NMDARs and σ1Rs and may improve the presumed hypofunction of NMDARs in patients with schizophrenia [6,10,16]. The protective effects of DHEA and DHEAS can also be attributed to their modulation of type A GABA receptors (GABA_A_Rs) and protection of mitochondria from intracellular Ca^2+^ overload [6]. DHEA and DHEAS also exhibit antioxidant and anti-inflammatory potential outside the CNS [5,17].

σ1Rs may also be involved in the pathogenesis of schizophrenia. It is therefore possible that increased concentrations of PREGS, DHEA, and DHEAS, which positively modulate σ1Rs, and/or decreased concentrations of P, which is an antagonist of σ1Rs, may contribute to schizophrenia. DHEA and DHEAS stimulate NMDAR activity via σ1Rs, and overactivation of σ1Rs can lead to NMDAR hyperactivity and subsequent reduction in NMDAR density, which has been reported in schizophrenia [6,7].

Besides NMDARs and GABA_A_Rs, PREGS and DHEAS modulate several types of ionotropic receptors, such as AMPARs, nicotinic receptors, melastatin receptors (TRPM3s), TRPC5, and vanilloid receptors (TRPV1s) and may improve cognitive function and counteract pain transmission and fear (see [1,18]). However, both DHEA and DHEAS support dopaminergic activity, which is an undesirable factor in schizophrenia.

Cortisol produced by the *zona fasciculata* of the adrenal cortex increases the readiness of the body in stressful situations by increasing blood sugar through gluconeogenesis and also suppressing the immune system [19]. In addition, cortisol influences the activity of several neurotransmitter systems that affect reward processing, attention regulation, executive function, mood, and emotion, such as serotonin, GABA_A_Rs, glutamate, dopamine, and acetylcholine [10]. Specifically, cortisol suppresses the synthesis, release, and metabolism of serotonin, which increases the risk of depression and also affects the release of excitatory amino acids [10]. Chronically increased cortisol secretion can lead to cognitive impairment [1,20,21]. In addition, patients with schizophrenia often exhibit an impaired multisystem response to stress with elevated morning cortisol levels, blunted cortisol response upon awakening, and reduced cortisol response to stress [21]. In contrast, DHEAS exerts an anti-glucocorticoid effect and protects the hippocampus from the damaging effects of cortisol [21,22,23]. 

The effects of P and especially its metabolites in the CNS, or its immunomodulatory effects, have also been reported in men [24]. These metabolites have antipsychotic effects, and their reduced concentrations can cause psychotic fragility. The antipsychotic effects of P likely result from the effects of steroids on the GABAergic and dopaminergic systems, with the P metabolite ALLO inhibiting dopaminergic neurotransmission [7,25]. Decreased levels of GABAergic ALLO, together with increased levels of antagonistic DHEA and DHEAS, may lead to the impaired GABAergic function that has been reported in association with schizophrenia and to excessive serotonergic activity. [6]. Testosterone (T) is the main male sex hormone affecting the density of dopaminergic neurons, and low T levels in men may be associated with cognitive impairment [5,26,27,28].

To summarize, steroids participate in the pathophysiology of schizophrenia. Except of our previous study, on different numbers of volunteers, with the absence of some important bioactive steroids and without an attempt to interpret steroidomic changes in terms of steroid molar ratios [15], a systematic study involving the vast majority of the human steroidome is still lacking. We have therefore attempted here to fill in this missing information in order to get an overall picture of the relationship between steroids and schizophrenia in men.

## 2. Results

Table 1 shows the discrimination between male schizophrenics and controls based on steroids using orthogonal prediction to latent structure (OPLS) and multiple regression (MR) analyses. Differences between steroid levels in controls and patients found by an ANOVA model (factor Schizophrenia) including multiple comparisons in Stage 1 and indication of relevant variables from OPLS models for Stage 1 are shown in Table 2. The results of the complete ANOVA model and are illustrated in Table S1. Table 3 shows changes in steroid molar ratios, which may reflect steroidogenic enzyme activities or the balances between steroidogenic enzymes in patients.. The individual OPLS sub-models for steroid levels, and also for groups of molar ratios that could reflect the activities of individual enzymes or balances between enzymes, are listed in Table S2. Table 4 presents significant Schizophrenia × Stage interactions, showing distinct changes between Stage 2 and Stage 1 for patients and controls for some steroids and molar ratios.

The symbols ↑ and ↓ in Table 1, Table 2, Table 3 and Table 4 and Tables S1 and S2 represent positive and negative correlations with schizophrenia, respectively. In other words, these are higher and lower levels, respectively, in patients compared to controls. Associations between the steroidome and schizophrenia were assessed in parallel using an ANOVA with multiple comparisons in Stage 1 (Table S1) and a partial OPLS models in Stage 1 (Table S2). Table S2 evaluated, separately, the effect of steroids and the effect of steroid groups in relation to the functioning of individual enzymes of steroidogenesis and the balances between these enzymes.

### 2.1. Discriminating Schizophrenics from Controls Based on Circulating Steroids

**Figure 1:**
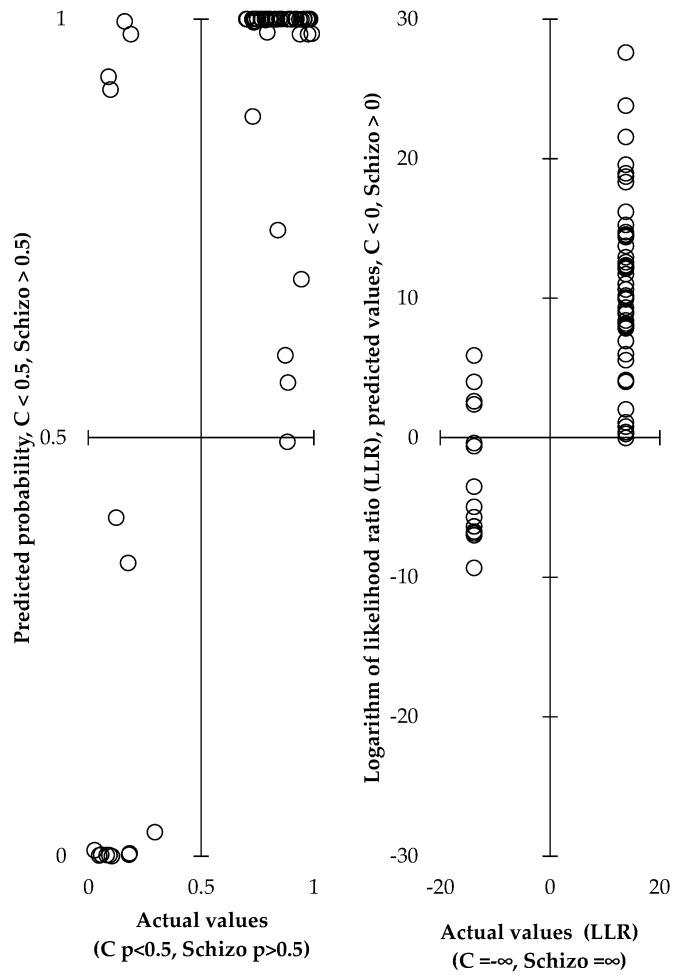
Discrimination between male schizophrenics in Stage 1 and controls based on steroids and steroid product to precursor ratios, as evaluated by an orthogonal predictions to latent structure (OPLS) model. Sensitivity = 0.923 (0.851–0.996), specificity = 0.917 (0.76–1), shown as means with 95% CI.

The results show significant steroidomic changes in men with schizophrenia, suggesting an important role for steroids in the pathophysiology of this disease and the potential to exploit these differences in its diagnosis. Based on all steroid levels and all molar ratios, we created an overall OPLS model that distinguishes between patients and controls (Table 1, Figure 1). This model, including 12 relevant steroids and 32 molar ratios, discriminates patients from controls with a sensitivity of 0.923 (0.851–0.996) and specificity of 0.917 (0.76–1).

### 2.2. Altered Steroid Levels in Schizophrenic Men

Of the 85 steroids, 38 are lower, 37 are not significantly different, and 10 are higher in patients compared with controls (*p* < 0.001, Mann–Whitney test). Thus, there is a predominant and significant trend towards lower steroid levels in patients. Of the 49 unconjugated steroids, 24 are lower, 21 are not significantly different, and 4 are higher in patients compared with controls (*p* < 0.001, Mann–Whitney test). So, as with all steroids studied, the prevailing trend towards lower levels of unconjugated steroids in patients is maintained. However, no significant trend was found for conjugated steroids in relation to schizophrenia. Of the 36 conjugated steroids, 14 are lower, 16 are not significantly different, and 6 are higher in patients compared with controls (*p* = 0.075, Mann–Whitney test), which is not enough to reach statistical significance.

While there is a significant trend towards lower levels of pregnanes (C21 steroids), as of 54 pregnanes, 31 levels are lower in patients, 20 levels are not significantly different, and 3 levels are higher compared to controls (*p* < 0.001, Mann–Whitney test), for androstanes (C19 steroids), there is no significant trend, as of the 31 androstane steroids, 7 levels are lower, 17 levels are not significantly different, and 7 levels are higher in patients compared to controls (*p* = 1, Mann–Whitney test).

#### 2.2.1. Δ^5^ and Δ^4^ Steroids

On the whole, there is no significant trend for Δ^5^ steroids in relation to schizophrenia, as of the 18 Δ^5^ steroids, 4 are lower, 12 are not significantly different, and 2 are higher in patients compared with controls, *p* = 0.429, Mann–Whitney test). However, the first steroid in the metabolic pathway of steroidogenesis, PREG, and its 20α-dihydrometabolite showed significantly lower levels in patients.

#### 2.2.2. Glucocorticoids (C21 Δ^4^ Steroids) and 11β-Hydroxy-androgens (C19 Δ^4^ and 5α/β Steroids)

Glucocorticoids and 11β-hydroxy-androstanes show a significant trend towards lower levels in patients, as of the 11 11β-hydroxy-steroids, eight are lower, two are not significantly different, and one is higher in patients compared with controls (*p* = 0.022, Mann–Whitney test).

#### 2.2.3. Progestogens

Of the seven progestogens (Δ^4^ pregnanes), four are lower, three are not significantly different, and none are higher in patients compared with controls (*p* = 0.056, Mann–Whitney test), which is not enough to reach statistical significance for their apparently reduced levels in patients, only to approach it.

#### 2.2.4. 5α-Reduced Steroids

Of the 28 5α-reduced steroids, 13 are lower, 10 are not significantly different, and 5 are higher in patients compared with controls (*p* = 0.061, Mann–Whitney test), which indicates predominantly lower levels in patients but below the level of statistical significance. However, this trend becomes statistically significant after excluding 5α-androstanes, as of the 17 5α-pregnanes, 10 are lower, 6 are not significantly different, and 1 is higher in patients compared with controls (*p* = 0.007, Mann–Whitney test).

For 5α-androstanes this trend is insignificant because of the 11 5α-androstanes, 3 are lower, 4 are not significantly different, and 4 are higher in patients compared to controls (*p* = 0.741, Mann–Whitney test).

#### 2.2.5. 5β-Reduced Steroids

As in the 5α-steroids, the 5β-steroids show predominantly but already significantly lower levels in patients, as of the 18 5β-reduced steroids, 7 are lower, 10 are not significantly different, and 1 is higher in patients compared with controls (*p* = 0.036, Mann–Whitney test). Similarly, as for the 5α-steroids, this trend is more significant after exclusion of 5β-androstanes, as of the 12 5β-pregnanes, 6 are lower, 6 are not significantly different, and none are higher in patients compared with controls (*p* = 0.016, Mann–Whitney test). Again, as in the case of the 5α-androstanes, this trend is insignificant for 5β- androstanes, as of the six 5β-androstanes, one is lower, four are not significantly different, and one is higher in patients compared with controls (*p* = 1, Mann–Whitney test).

#### 2.2.6. Androstenedione and Active Androgens

While the T precursor A in the Δ^4^ pathway showed elevated levels in patients, there are lower levels of both unconjugated T and conjugated T, as well as 5α-DHT. However, the levels of conjugated 5α-DHT are elevated.

### 2.3. Changes in the Steroid Molar Ratios

To provide a more detailed picture of the changes in steroidogenesis, the values of the respective steroid molar ratios were evaluated.

#### 2.3.1. The Overall Conversion of 17-Deoxy-pregnanes to Corresponding Androstanes

The general trend of molar ratios, which may reflect the overall functioning of CYP17A1 (hydroxylase + lyase step), was towards increased conversion of 17-deoxy-pregnanes to the corresponding androstanes, as of the 23 molar ratios describing the conversion of 17-deoxy-pregnanes to the corresponding androstanes, 1 is lower, 7 are not significantly different, and 15 are higher in patients compared to controls (*p* < 0.001, Mann–Whitney test).

#### 2.3.2. The Conversion of 17-Deoxypregnanes to Corresponding 17-Hydroxy-pregnanes

The molar ratios of the steroids, which may reflect the functioning of CYP17A1 in the hydroxylase step, show no significant trend for the conversion of C17-deoxy-pregnanes to their 17-OH-metabolites, as of the 16 molar ratios, 4 are lower, 9 are not significantly different, and 3 are higher in patients compared to controls (*p* = 0.727, Mann–Whitney test). However, it should be emphasized that the ratios of 17-OH-PREG/PREG and 17-OH-PREGS/PREG are considerably higher in patients, demonstrating higher 17-hydroxylation in the key Δ^5^ pathway, notwithstanding the insignificant general trend.

#### 2.3.3. The Conversion of 17-Hydroxy-pregnanes to Corresponding Androstanes

The general trend in the conversion of 17-OH-pregnanes to androstanes that may reflect functioning of CYP17A1 lyase step for both classical (“frontdoor”) and “backdoor” pathways is similar to increased conversion of 17-deoxy-pregnanes to the corresponding androstanes in patients. Of the 16 molar ratios, none are lower, 4 are not significantly different, and 12 are higher in patients compared to controls (*p* < 0.001, Mann–Whitney test).

#### 2.3.4. The Conversion of Δ^5^ Steroids to Their Δ^4^ Counterparts

Steroid molar ratios that may reflect functioning of HSD3Bs (type 1 and 2 3β-hydroxysteroid dehydrogenases) show no consistent trend in relation to schizophrenia, as of the 12 molar ratios describing the conversion of Δ^5^ steroids to their Δ^4^ counterparts, 3 are lower, 7 are not significantly different, and 2 are higher in patients compared with controls (*p* = 0.687, Mann–Whitney test). However, despite the lack of a general prevailing trend, two molar ratios in the key cortisol pathway (17-OH-P/17-OH-PREG and 17-OH-P/17-OH-PREGS) are significantly lower in patients when compared to controls.

#### 2.3.5. The Balances between Sulfated and Unconjugated Steroids

Of the 32 molar ratios describing the ratio of conjugated steroids to unconjugated steroids, none are lower, 17 are not significantly different, and 15 are higher in patients compared to controls (*p* < 0.001, Mann–Whitney test), indicating increased steroid sulfation in patients.

#### 2.3.6. 11β-Hydroxylation of Androstane Steroids

The all investigated steroid molar ratios that may reflect CYP11B1 functioning are lower in patients.

#### 2.3.7. 7α-, 7β-, and 16α-Hydroxylation of Δ^5^ Androstanes

Of the eight molar ratios describing the functioning of 7α-, 7β- and 16α-hydroxylation enzymes (CYP7B1, CYP3A4, CYP3A7), none are significantly lower, four are not significantly different, and four are higher in patients compared to controls (*p* = 0.054, Mann–Whitney test), which, although not enough to reach statistical significance with such a small number, is close to it, indicating increased potency of these enzymes in patients.

#### 2.3.8. Steroid Molar Ratios Probably Related to HSD11B1

Of the four molar ratios describing the functioning of HSD11B, none are lower, three are not significantly different, and one is higher in patients compared with controls (*p* = 0.453, Mann–Whitney test). Given the low number of these molar ratios, no definitive conclusions could be drawn regarding the overall trend in relation to schizophrenia.

#### 2.3.9. Steroid Molar Ratios Probably Related to 5α-Reductases (SRD5A1 and SRD5A2)

Of the 21 described molar ratios that may reflect the functioning of 5α-reductases, 5 are lower, 8 are not significantly different, and 8 are higher in patients compared to controls (*p* = 0.416, Mann–Whitney test), suggesting the absence of a significant trend in relation to schizophrenia.

#### 2.3.10. Steroid Molar Ratios Probably Related to AKR1D1

Of the 21 molar ratios that may reflect 5β-reductase (AKR1D1) functioning, 2 are lower, 16 are not significantly different, and 3 are higher in patients compared to controls (*p* = 0.672, Mann–Whitney test), suggesting the absence of a significant trend in relation to schizophrenia, as in the case of 5α-reductases.

#### 2.3.11. Steroid Molar Ratios Probably Related to a Balance between AKR1C1 and HSD17B2

Of the 19 molar ratios that may reflect a balance between AKR1C1 and HSD17B2, 3 are lower, 11 are not significantly different, and 5 are higher in patients compared with controls (*p* = 0.494, Mann–Whitney test), suggesting the absence of a significant trend in relation to schizophrenia.

#### 2.3.12. Steroid Molar Ratios Probably Related to a Balance between AKR1C2 on One Side and HSD17B2 and HSD17B2 on the Other

Of the 26 molar ratios that may reflect a balance between AKR1C2 on one side and HSD17B2 and HSD17B6 on the other, 7 are lower, 13 are not significantly different, and 6 are higher in patients compared with controls (*p* = 0.792, Mann–Whitney test), suggesting the absence of a significant trend in relation to schizophrenia.

#### 2.3.13. Steroid Molar Ratios Probably Related to Balance between AKR1C3 and HSD17B2

Of the 16 molar ratios which may reflect the balance between HSD17B3 and AKR1C3 on the one hand and HSD17B2 on the other, 10 are lower, 5 are not significantly different, and 1 is higher in patients compared to controls (*p* = 0.007, Mann–Whitney test), suggesting a significant trend towards blunted conversion of 17-oxo androstanes to their 17β-hydroxy counterparts in the context of schizophrenia.

### 2.4. Effect of Antipsychotic Treatment

Our data show that the steroidomic changes are not much affected by antipsychotic treatment, and if so, the effects were not consistent (Table 4).

## 3. Discussion

In terms of the significance and interpretability of the results in relation to the pathophysiology of schizophrenia and perhaps for future diagnostic use, the assessment of steroid molar ratios may be of greater importance than the monitoring of individual steroids. The appropriate molar ratios of steroids could be related to the functioning of specific steroidogenic enzymes or to the balance between them. Therefore, in addition to steroid levels, we also assess changes in the appropriate molar ratios in relation to schizophrenia.

Firstly, we focus on the comparison of our current results with the results of our previous study and the studies of other authors. Most studies examining changes in circulating steroid levels in relation to schizophrenia involve one or a few steroids and do not address changes in the broader context of steroid metabolism. Moreover, data aimed at qualitatively estimating changes in steroidogenic enzyme functioning based on steroidomics in patients with schizophrenia are still very rare, and systematic studies in this regard are almost entirely absent. Regarding multi-steroid studies of schizophrenia, our 2011 (18 5α/β-reduced steroids) [29] and 2013 studies (39 steroids) [15]) based on the GC-MS/MS platform should be referenced.

In our 2013 study, we evaluated the levels of 22 unconjugated and 17 conjugated steroids on a GC-MS platform in 22 patients with drug-naive (first-episode) schizophrenia (13 men and 8 women) before and after 6 months of treatment with atypical antipsychotics and compared the results with matched controls (22 men, 25 women) [15].

Further study of this kind includes more recent data by Cai et al. [30] based on enzyme immunoassays (six steroids and eight molar ratios) after overnight fasting from 53 patients who were recruited in the first-episode psychosis stage of disease (FEPs). In addition, 43 age- and gender-matched HCs were included in this study. The patients were treated with antipsychotic drugs such as risperidone, olanzapine, quetiapine, aripiprazole, and haloperidol, and about 81% of patients were treated with risperidone either as a single drug (74%) or as an adjunctive (26%). The samples were collected in the same patient individuals at 1, 6 and 12 months after initiation of the treatment [30].

Most of the steroids investigated in the present study have not yet been studied in the association with schizophrenia. Therefore, we focused on those that have been reported in the literature in the context of schizophrenia. Our current data show lower levels of PREG in the patients, which is in accordance with our previous study [15] and studies by Ritsner et al. [31] and McKenzie et al. [7]; although, in the latter case the difference between patients and controls did not reach statistical significance. However, Cai et al. reported higher [30] levels of PREG in patients. Concerning the levels of PREGS, our current data show no significant difference between patients and controls, while our previous study reported higher levels of PREGS in patients compared to controls [15]. Our current data show lower levels of both 20α-dihydro-PREG and 20α-dihydro-PREGS in patients compared to controls, whereas our previous study found no significant difference for either steroid [15]. 17-OH-PREG showed no significant difference between patients and controls either in the present study or in our previous study [15].

For DHEA levels, we found no significant difference between patients and controls, which is similar to Ritsner et al. in their 2004 study [22]. In addition, the meta-analysis by Misiak et al. reported an insignificant difference for DHEA [19]. On the other hand, Ritsner et al. in a later study (2007) reported higher DHEA levels in patients [31], which is similar to the recent study by Ji et al. [32]. Regarding DHEAS, we found no significant difference between patients and controls, as in our previous study [15]. On the other hand, Peng and Li [33] and Dogan Bulut et al. [9] reported higher levels of DHEAS in patients, which is similar to the result of the meta-analysis by Misiak et al. [19]. In the case of ADIOL, we found no significant difference between patients and controls in this study, as in our previous study [15].

In contrast to the results of the previous study, where we found no significant difference between male patients and controls for T [15], our current data show lower T levels in patients versus controls, which is consistent with other studies [19,34]. Both our current data and data from our previous study show higher A values in male patients compared to controls [15].

For cortisol, we found no significant difference between patients and controls in the present study, which is in contrast to our previous study and other studies where cortisol levels were higher in patients [9,33,35].

Progesterone levels in our current study showed no significant difference between patients and controls, which is similar to the study by Cai et al. [30]. However, in our previous study, progesterone levels were higher in patients [15]. Although we found no significant difference in ALLO levels between patients and controls in our previous study [15], in the present study we found lower ALLO levels in patients compared to controls, which is similar to the study by Cai et al. [30].

Next, the ability of steroids and steroid molar ratios to distinguish patients from controls was assessed using multivariate regression with dimensionality reduction (an OPLS model). Based on the levels of all steroids and all molar ratios, we built an OPLS model discriminating between patients and controls. This model, including 12 relevant steroids and 32 molar ratios discriminated patients from controls with a sensitivity of 0.923 (0.851–0.996) and specificity of 0.917 (0.76–1), which was comparable to the results of our earlier study [15]. Our previous study included substantially lower number of male patients (*n* = 13) and a slightly higher number of male controls (*n* = 22); our current study includes 51 male patients and 16 male controls.

Further, we investigated whether there is a significant overall trend in changes in steroid levels in patients compared to controls. Due to the number of steroids determined, we were able to evaluate the prevailing trends in their aggregate as well as for individual steroid subgroups. The aggregated results show a significant trend towards lower steroid levels in patients compared to controls. Given that, with the exception of the male gonads, most steroids in both sexes are synthesized either directly in the adrenal glands or from adrenal precursors in other peripheral tissues or directly in peripheral tissues [36], these results suggest an overall trend toward impaired adrenal activity in male patients with schizophrenia. This may have implications in terms of reduced production of mostly protective adrenal steroids, but also reduced biosynthesis of their GABAergic metabolites and immunoprotective 7α/β-,16α-hydroxy-C19 ∆^5^ androgens.

**Figure 2:**
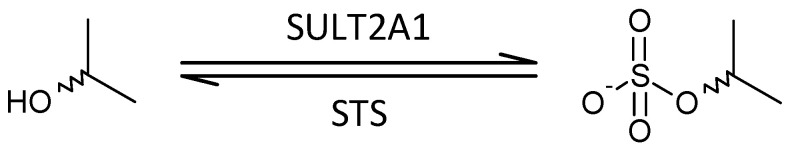
Scheme of the balance between steroid sulfotransferase 2A1 (SULT2A1) and steroid sulfatase (STS).

Our current study also addresses the question of whether there is a pathophysiologically relevant shift in the balance between unconjugated and sulfated steroids. Lower levels of PREG with unchanged levels of its sulfate as well as increased conjugated/unconjugated steroid ratio (C/U) for PREG in patients compared to controls suggest higher sulfotransferase (SULT2A1) functioning in the *zona fasciculata* of the adrenal gland, where C21 steroids are produced, and/or in the *zona reticularis*, where C19 steroids are primarily produced. Nevertheless, the *zona reticularis* is still in contact with circulating unconjugated PREG. In addition, SULT2A1 functioning of the *zona reticularis* is several times higher compared to the *zona fasciculata* [37]. A second possibility could be reduced sulfatase (STS) functioning, which would maintain a higher cholesterol sulfate to cholesterol ratio and also prevent desulfation in downstream steroidogenic pathways [38]. In this context, it should be noted that cholesterol sulfate can be directly converted to PREGS [39], and PREGS can also be directly converted to 17-OH-PREGS. In contrast, a direct conversion of 17-OH-PREGS to DHEAS does not occur [40]. As for the tissue in which PREG sulfation predominantly occurs, the most likely is the *zona fasciculata* in the adrenal cortex, which (due to the absence of CYB5) cannot convert pregnanes to androstanes but can convert pregnanes, such as PREG and 17-OH-PREG, to their sulfates ([37]). A simplified scheme of the balance between unconjugated and sulfated steroids is shown in Figure 2.

As already mentioned, both PREGS and DHEAS are modulators of several types of ionotropic receptors, such as NMDARs, AMPARs, nicotinic receptors, melastatin receptors (TRPM3s), TRPC5, or vanilloid receptors (TRPV1s), and may improve cognitive function while counteracting pain and fear transmission (see [1,18]). Therefore, increased PREG sulfation may protect patients from deficiency of PREGS, which is a glutamatergic steroid. This could be important because NMDAR hypofunction is common in patients with schizophrenia and pain and fear are closely associated with stress, which is considered an important target for antipsychotic treatment [6,10,16,41]. In addition, PREGS is a pro-cognitive steroid, which, besides positive modulation of certain subtypes of NMDARs and negative modulation of GABA_A_Rs and glycine receptors is also neuroprotective via inhibition of AMPAR (summarized in [18]). Moreover, PREGS is a negative modulator of L-type VGCC, which specific mutations are amongst the most consistently detected genetic risk factors in schizophrenia [42]. Our data suggest that although increased PREG sulfation may inhibit cortisol synthesis, it may in turn contribute to the maintenance of PREGS levels with its beneficial effects in relation to schizophrenia.

In general, the balance between sulfated and unconjugated steroids, expressed in terms of molar ratios of unconjugated to conjugated forms of steroids (C/U), was clearly shifted to higher values in patients. Increased C/U was observed for PREG and most Δ^4^ steroids and also for 5α/β reduced steroids, but not for any other Δ^5^ steroid.

The last and most important of the enzymes in the cortisol pathway is CYP11B1, converting inactive 11-deoxycortisol to active cortisol. Therefore, we investigated changes reflecting the functioning of this enzyme in relation to schizophrenia. Our present data show that most 11β-hydroxy-steroids, to which corticosteroids belong, showed a significant trend towards lower levels in patients, which may be a consequence of lower CYP11B1 functioning. CYP11B1 forms 11β-hydroxy-steroids from their 11-deoxy-precursors. An alternative route of 11β-hydroxy-androgen synthesis could also be the cleavage of the corticoid side chain in the CYP17A1 lyase step. However, cortisol and its 5α/β-reduced metabolites are not preferred substrates for conversion to 11β-hydroxy-androgens; therefore, they contribute only negligibly to this conversion [43,44,45].

**Figure 3:**
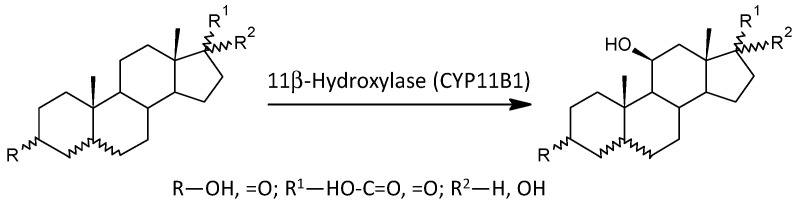
Scheme of the actions of 11β-hydroxylase (CYP11B1).

As can be seen from the molar ratios of 11β-hydroxy-steroids to their corresponding 11-deoxy-counterparts and the overall trend of 11β-hydroxy-steroids (both significantly lower in patients), the last metabolic step in cortisol synthesis is also blunted in male schizophrenics. Aside from conversion of 11-deoxycortisol to cortisol, CYP11B1 simultaneously converts 11-deoxy-androstanes to 11β-hydroxy-androstanes. A simplified scheme of the action of CYP11B1 is shown in Figure 3.

Next, we focused on possible changes in the levels of GABAergic 5α/β-reduced pregnanes and androstanes in patients compared to controls. Levels of pregnane steroids tend to be lower in patients, especially in unconjugated 5α/β-reduced pregnanes including the most potent of them ALLO and pregnanolone, but also the levels of some 5α/β-reduced androstanes. Given that the 3α-OH-5α/β-pregnanes and androstanes are GABAergic neuroinhibitory and neuroprotective agents, this trend may indicate attenuation of neuroprotection in patients. As suggested above, the lower levels of 5α/β-reduced pregnane may be mainly due to the generally suppressed adrenal steroid production in patients.

**Figure 4:**
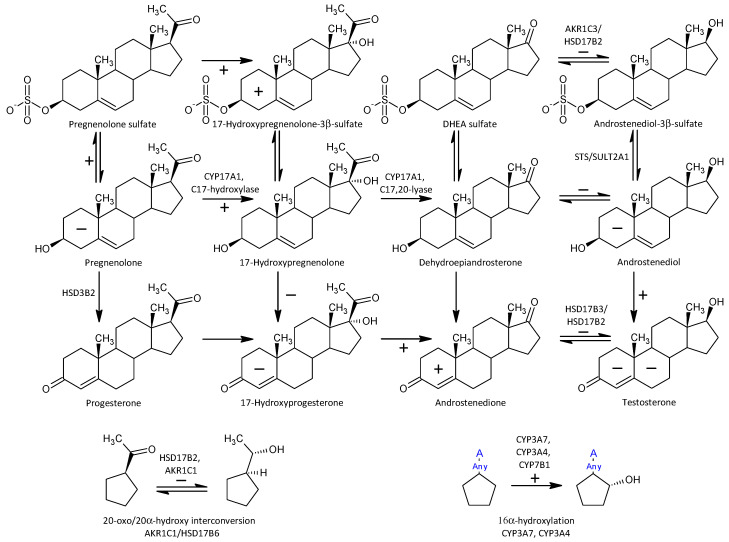
Simplified scheme of steroidogenesis in the Δ^5^ and Δ^4^ pathways; the symbols + and − indicate higher and lower steroid levels, respectively, in Stage 1 patients (compared to controls). The location of these symbols in the steroid A and B rings refer to unconjugated and conjugated steroids, respectively. The location of the + and − symbols on the arrows indicating steroid conversion refers to a higher and lower molar ratio of product to precursor in patients (compared to controls), respectively.

One of the important objectives of this study was to observe possible alterations in the conversion of 17-deoxy-pregnanes to 17-hydroxy-pregnanes and further to androstanes. Our current results show a consistent, highly significant trend to increased conversion of 17-deoxy-steroids to the corresponding androstanes in patients compared to controls. This is consistent with the results by Ritsner et al. [13], reporting a higher molar ratio of DHEA/PREG in patients with schizophrenia compared to controls. These authors also hypothesized that an increase in the functioning of CYP17A1, which converts PREG to DHEA, might be involved [13]. However, Ritsner et al. did not monitor 17-OH-PREG levels, and therefore it was not possible to infer from their results whether the CYP17A1 hydroxylase step, the lyase step, or both were involved in this finding. Our comprehensive data involving a series of 17-deoxy-pregnans and the corresponding 17-hydroxy-pregnans and androstanes allow such a consideration, as will be discussed below (a simplified scheme of steroidogenesis in the Δ^5^ and Δ^4^ pathways is shown in Figure 4).

Although our present aggregated results do not show a significant trend for the conversion of 17-deoxy-pregnanes to 17-hydroxy-pregnanes, the ratios of 17-OH-PREG/PREG and 17-OH-PREGS/PREG are significantly higher in patients. This indicates a significantly higher conversion of 17-hydroxy-pregnanes to corresponding 17-hydroxy-preganes in the key Δ^5^ pathway, notwithstanding insignificant trends in the Δ^4^ and “backdoor pathway”. The general trend showing increased conversion of 17-hydroxy-pregnanes to the corresponding androstanes in patients was similar to the situation for the overall conversion of 17-deoxy-pregnanes to the corresponding androstanes. However, the situation in the Δ^5^ pathway was again different from that in the Δ^4^ and “backdoor” pathways, as the ratios of DHEA/17-OH-PREGS and DHEA/17-OH-PREGS did not differ between controls and patients.

**Figure 5:**
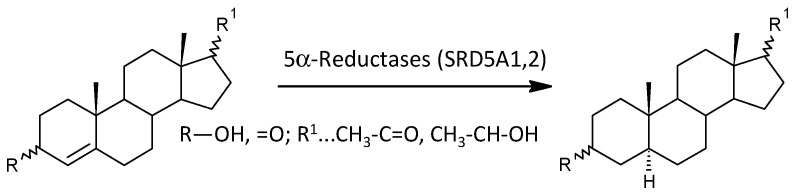
Scheme of the actions of 5α-reductases (SRD5A1 and SRD5A2).

**Figure 6:**
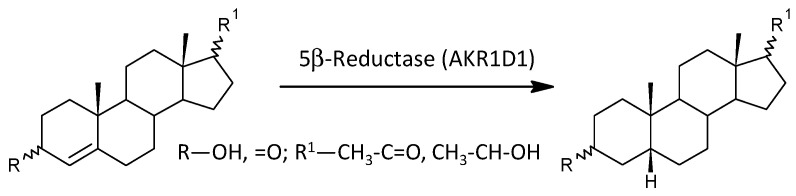
Scheme of the actions of a 5β-reductase (AKR1D1).

In view of the previous results, it is interesting that 17-hydroxy-ALLO is rapidly converted to androsterone (3α,5α-THA) by CYP17A1-lyase, even in the absence of CYB5, and that 17-hydroxy-ALLO is also a better substrate for CYP17A1-lyase than 17-OH-PREG [46]. Although CYB5, which activates the CYP17A1 lyase step, has low tissue specificity, its expression in the adrenal cortex is about four times higher than in most other tissues (http://biogps.org/#goto=genereport&id=80777, accessed on 26 June 2024). In contrast, CYP17A1 is more than 1500 times more expressed in the adrenal cortex compared to most other tissues and about 70 and 40 times more expressed in the kidneys and testes, respectively (http://biogps.org/#goto=genereport&id=1586, accessed on 26 June 2024). While the formation of Δ^5^ steroids occurs mainly in the adrenal cortex, 5α/β-reduced steroids are mainly formed extra-adrenally. From the enzymes forming the 5α/β-reduced steroids, SRD5A1 is tissue non-specific (http://biogps.org/#goto=genereport&id=6715, accessed on 26 June 2024), and SRD5A2 has low tissue specificity with about 3-fold higher expression in the liver compared to other tissues (http://biogps.org/#goto=genereport&id=6716, accessed on 26 June 2024). Both isoforms of SRD5A convert Δ^4^ steroids to their 5α-reduced counterparts (Figure 5). AKR1D1, converting Δ^4^ steroids to their 5β-reduced counterparts, is liver specific with about 60-fold higher expression in the liver compared to other tissues (http://biogps.org/#goto=genereport&id=6718, accessed on 26 June 2024) (Figure 6).

These data indicate that extra-adrenal conversion to 17-deoxy-pregnanes to androstanes may be independent of the CYB5 enzyme, which is under-expressed outside the adrenal *zona reticularis* [37]. This may be the reason why, in the synthesis of Δ^5^ androstanes (which make up the bulk of gonadal T precursors), the adrenal cortex prefers the Δ^5^ pathway, while other tissues, especially the liver, prefer the synthesis of 5α/β-reduced androgens independent of CYB5. Indeed, as our results show, circulating levels of intermediates (17-OH-ALLO and 17-OH-pregnanolone), on the path from 17-deoxy-5α/β-reduced pregnanes to 5α/β-reduced androstanes, are disproportionately lower compared to the latter steroids, particularly the sulfated ones.

We also found increased levels of 17-OH-PREGS in patients, which in addition to likely increased SULT2A1 functionality, could indicate increased conversion of PREGS to 17-OH-PREGS, which is a possible metabolic step as documented in the literature [40]. Another explanation could be a blocked pathway from 17-OH-PREG/S to downstream steroids. Nevertheless, both alternatives indicate an abnormality in cortisol synthesis, despite the fact that cortisol levels are not significantly altered in patients compared to controls.

**Figure 7:**
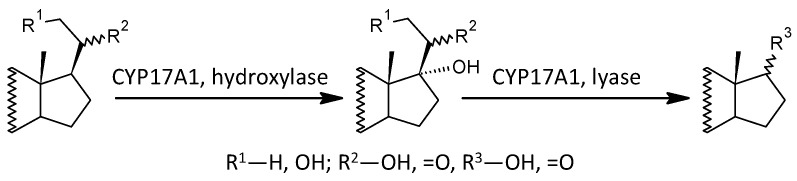
Scheme of the actions of C17-hydroxylase, C17,20-lyase (CYP17A1).

**Figure 8:**
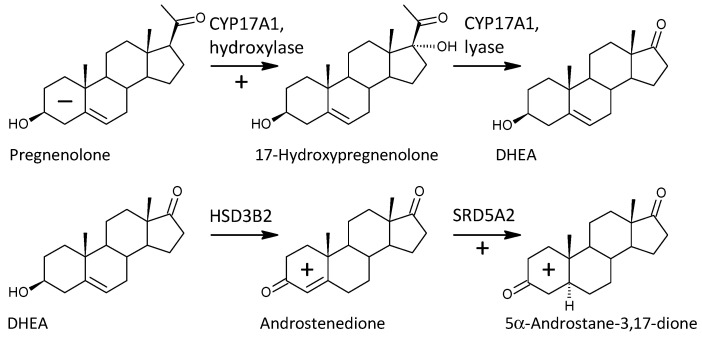
Simplified scheme of classical “frontdoor” pathway in the synthesis of 5α-reduced C19 steroids; The symbols and their placement have been explained in the legend to Figure 4.

**Figure 9:**
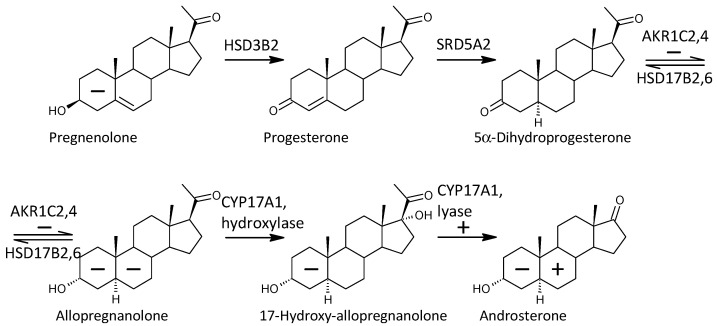
Simplified scheme of “backdoor” pathway in the synthesis of 5α-reduced C19 steroids; the symbols and their placement have been explained in the legend to Figure 4.

In summary, the conversion of 17-deoxy-pregnanes to the corresponding androstanes in patients was enhanced in the Δ^5^, Δ^4^, and “backdoor” pathways. In the case of the Δ^5^ pathway, this was due to increased conversion of 17-deoxy-pregnanes to the corresponding 17-hydroxy-pregnanes (pregnenolone to 17-hydroxypregnenolone). In the Δ^4^ and “backdoor” pathways, however, the conversion of 17-deoxy-pregnanes to the corresponding androstanes was provided by the increased conversion of 17-hydroxy-pregnanes to the corresponding androstanes. The illustration of conversion of 17-deoxy-pregnanes to 17-hydroxypregnanes and 17-pregnanes to androstanes are in Figure 7, Figure 8 and Figure 9.

**Figure 10:**
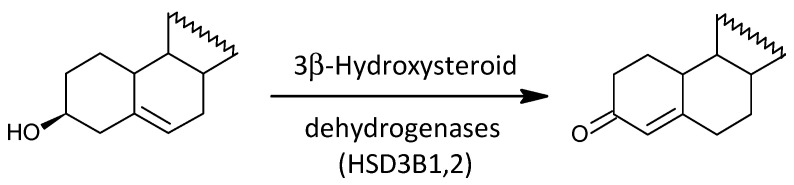
Scheme of the actions of 3β-hydroxysteroid dehydrogenases (HSD3B1,2).

The conversion of 17-OH-PREG to 17-OH-P is an important metabolic step, which was also investigated in the present study (Figure 10). To the best of our knowledge, there is no mention in the literature of the relationship of this metabolic step to schizophrenia. Although molar ratios that may reflect HSD3B functioning do not show a significant consistent trend in our present results in relation to schizophrenia, molar ratios in the key pathway leading to cortisol synthesis are significantly lower (17-OH-P/17-OH-PREG and 17-OH-P/17-OH-PREGS) in patients compared to controls. In addition, the levels of 17-hydroxy-progestogens were reduced. The above data suggest suppressed adrenal HSD3B2 function (expressed in the *zona fasciculata*) and also a bottleneck in cortisol synthesis, although circulating levels of this active glucocorticoid do not differ between patients and controls.

Of the remaining molar ratios reflecting HSD3Bs functioning, P/PREG and A/DHEA were unchanged and the T/ADIOL ratio was increased. The last data may suggest an increased conversion of inactive or mildly pro-estrogenic ADIOL to a key male hormone.

**Figure 11:**
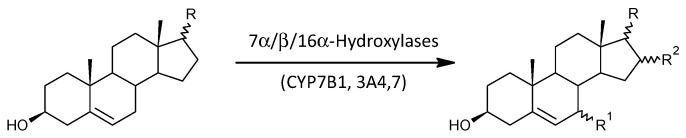
Scheme of 7α/β/16α-hydroxylation of the Δ^5^ C19 steroids.

In this study, we also investigated whether there is any trend towards altered synthesis of the immunoprotective 7α/β-,16α-hydroxy-C19 ∆^5^ androstanes in patients. The simplified scheme of 7α/β/16α-hydroxylation of the Δ^5^ C19 steroids is shown in Figure 11. Only a borderline tendency towards increased 7α/β-,16α-hydroxylation of C19 ∆^5^ steroids was observed, probably due to the low number of corresponding molar ratios. The abovementioned metabolic steps are catalyzed by CYP7B1, CYP3A4, and CYP3A7 enzymes. In general, the C19 Δ^5^ steroids (including their 7α/β-,16α-hydroxy-metabolites) mitigate the severity of autoimmune diseases [47,48,49,50,51,52], and on the other hand, autoimmune diseases may suppress the production of adrenal C19 Δ^5^ steroids [47,53]. The C19 Δ^5^ steroids and their 7α/β-,16α-hydroxylated metabolites may also counteract the suppression of the primary immune response by glucocorticoids [54]. DHEA controls the Th1/Th2 balance and either favors the Th1 component or attenuates the production of both components [51,55]. The C19 Δ^5^ steroids also suppress cell-mediated immunity and the formation of autoantibodies [49,50,51,52,56], and they may induce restoration of the Th1-dominated cytokine profile. Based on the above data, we hypothesize that increased 7α-, 7β-, and 16α-hydroxylation plays a role in the transition from adaptive immunity involving autoimmunity to the innate immune system and is related to inflammatory processes that may accompany schizophrenia [1]. However, synthetic anti-inflammatory derivatives of 5-androstene-3β,7β,17β-triol also suppress the production of inflammatory markers such as C-reactive protein, interleukin 17 (IL-17), TNFα, and interleukin 6 (IL-6) signaling, as well as the expression of mRNA for IL-6 and matrix metalloproteinase in inflamed tissues. In addition, they also suppress pro-inflammatory cytokines in the lung and intensely stimulate splenic regulatory T cells [57].

The first mechanism that may explain the immunomodulatory effects of 7α/β-hydroxy-∆^5^-androstanes is based on data showing that the autoimmune response can also be induced by estradiol, specifically via estrogen receptors, and that catabolism of C19 estrogen precursors via 7α/β/16α-hydroxylation can reduce estradiol levels [58]. Interestingly, estradiol stimulates CYP7B1 catalytic activity, mRNA, and the human CYP7B1 reporter gene in human embryonic kidney (HEK293) cells, and thus may feedback regulate DHEA, estradiol, and ADIOL levels in human tissues [59]. The ADIOL catabolite 5-androstene-3β,7α,17β-triol, which can be formed either by interconversion from 5-androstene-3β,7α,17β-triol or directly from ADIOL by the catalytic action of CYP3A4 and CYP3A7, is itself immunoprotective despite its low concentrations and high metabolic turnover [60]. Thus, the borderline tendency toward increased 7α/β-,16α-hydroxylation may counteract the development of both autoimmune and inflammatory complications that often accompany schizophrenia.

We also examined an important metabolic step that establishes the balance between the active glucocorticoid cortisol and its inactive metabolite cortisone. This balance is regulated by the reductive enzyme HSD11B1 (in the direction of cortisol) and the oxidative enzyme HSD11B2 (in the direction of cortisone). Glucocorticoids play a decisive role in the regulation of the immune system and act through binding to the GR. Although glucocorticoids are mainly a product of the adrenal *zona fasciculata*, they can be produced extra-adrenally, for example in cells of the immune system, intestine, skin, or brain [61].

**Figure 12:**
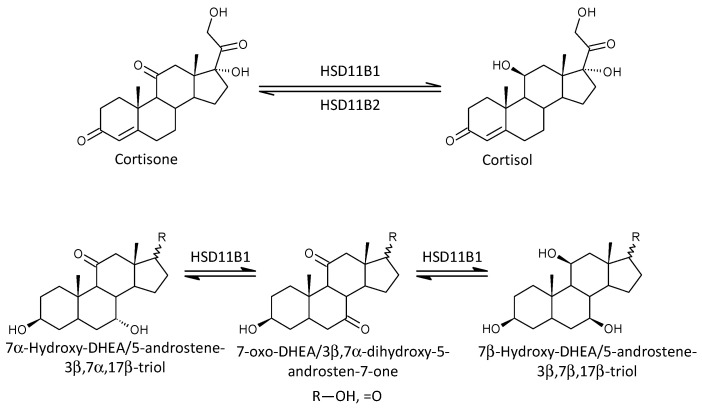
Scheme of action of type 1 and type 2 11β-hydroxysteroid dehydrogenases (HSD11B1 and HSD11B2, respectively).

A scheme of the balance between HSD11B1 and HSD11B2 is shown in Figure 12. HSD11B1 and HSD11B2 have distinct tissue expression patterns and contribute differently to circulating and local cortisol levels. While HSD11B1 is more widespread and mainly involved in cortisol activation, HSD11B2 focuses on cortisol inactivation in specific tissues. The enzyme is localized in selective tissues so that it can act as a paracrine or autocrine protector of the receptor against the action of the active form of glucocorticoid [62]. The above data suggest that HSD11B1 may be more important for the balance between cortisol and cortisone in the circulation than HSD11B2 [63]. Expression of mRNA for HSD11B1 is orders of magnitude higher in some specific tissues compared to median tissue expression, specifically in the liver (78×), adipocytes (17×), and smooth muscle (14×) (http://biogps.org/#goto=genereport&id=3290, accessed on 26 June 2024). Similarly, the mRNA expression for HSD11B2 is one to two orders of magnitude higher in some specific tissues compared to the median tissue expression, namely the colon (136×), kidney (75×), and salivary glands and small intestine (11×) (http://biogps.org/#goto=genereport&id=3291, accessed on 26 June 2024).

The second mechanism explaining the immunomodulatory effects of 7α/β-hydroxy-∆^5^-androstanes may be related to competition of these steroids for active sites on HSD11B1. As mentioned above, this enzyme catalyzes the conversion of inactive 11-oxo-glucocorticoids to their immunosuppressive 11β-hydroxy counterparts [64,65]. Although of the four molar ratios that may reflect HSD11B1 functioning, only one showed elevated values in patients; decreased cortisone levels were found when cortisol was unchanged.

**Figure 13:**
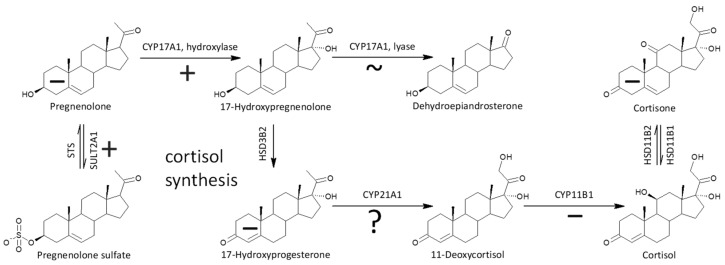
Simplified scheme of changed cortisol synthesis in patients; the symbols +, ~, and − signify higher, unchanged, and lower steroid levels or an activated, unchanged, or suppressed metabolic step, respectively, in schizophrenic males (compared to controls), respectively; the symbol “?” means that the relevant information is not available in our data.

**Figure 14:**
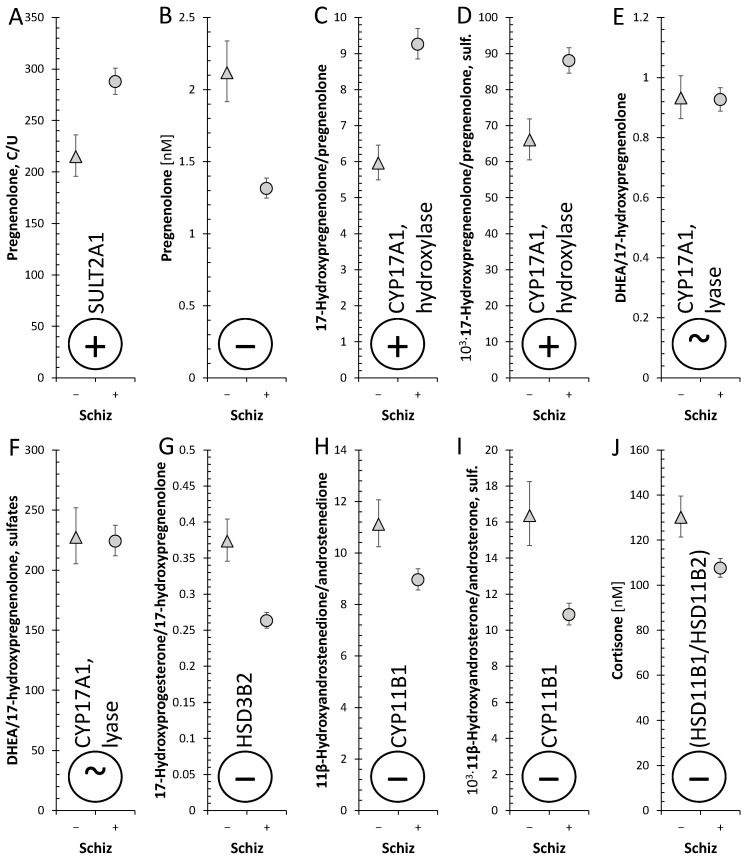
ANOVA model for evaluation of the alterations in cortisol biosynthesis and metabolism in patients with schizophrenia (Schiz), Age, and Stage of the experiment, including Schiz × Stage interaction. Schiz (only the effects of schizophrenia are shown). (**A**): F = 15.8, *p* < 0.001, η_p_^2^ = 0.217; (**B**): F = 35.6, *p* < 0.001, η_p_^2^ = 0.368; (**C**): F = 44.2, *p* < 0.001, η_p_^2^ = 0.424; (**D**): F = 20, *p* < 0.001, η_p_^2^ = 0.247; (**E**): F = 0, *p* = 0.92, η_p_^2^ = 0.000171; (**F**): F = 0, *p* = 0.87, η_p_^2^ = 0.00045; (**G**): F = 32.7, *p* < 0.001, η_p_^2^ = 0.368; (**H**): F = 10.5, *p* = 0.002, η_p_^2^ = 0.146; (**I**): F = 23.8, *p* < 0.001, η_p_^2^ = 0.284; (**J**): F = 11.3, *p* = 0.001, η_p_^2^ = 0.171; F—F-statistics, *p*—*p*-value, η_p_^2^—effect size; interpreting η_p_^2^ (Cohen): (0.01~small, 0.06~medium, >0.14~large). The triangles and circles with error bars represent re-transformed means with 95% confidence intervals for controls and patients, respectively; C—symbolizes conjugated steroids. The symbols +, ~, and − signify higher, unchanged, and lower steroid levels or an activated, unchanged, or suppressed metabolic step, respectively, in first-episode schizophrenic males (compared to controls); C/U symbolizes the ratio of conjugated to unconjugated steroid.

Although circulating cortisol did not differ between patients and controls, our current data showed significant changes in the pathway from PREG to cortisol as well as a shifted balance between bioactive cortisol and inactive cortisone in patients (see Figure 13 and Figure 14). These alterations can be summarized as follows.

First, the increased sulfation of PREG observed in patients with schizophrenia may be associated with reduced PREG levels and may negatively affect cortisol synthesis, as PREG is the primary steroid precursor for cortisol synthesis.

Second, the molar ratios 17-OH-PREG/PREG and 17-OH-PREGS/PREG were significantly higher in the patients, indicating higher conversion of 17-deoxy-pregnanes to their 17-hydroxy-couterparts in the key Δ^5^ pathway (but unaltered conversion of 17-OH-PREG to DHEA).

Third, the molar ratios in the key cortisol pathway related mainly to HSD3B2 were pronouncedly lower (17-OH-P/17-OH-PREG and 17-OH-P/17-OH-PREGS).

Fourth, the levels corticoids and 11β-hydroxy-androgens, as well as molar ratios of 11β-hydroxy-androstanes to their 11-deoxy precursors showed a significant trend toward lower values in patients, suggesting dampened CYP11B1 functioning.

Fifth, of the four molar ratios probably reflecting the balance between HSD11B1 and HSD11B2 only one showed elevated values in patients; however, the patients also had reduced cortisone levels with unchanged cortisol, suggesting either a shift from the inactive cortisone to the bioactive cortisol or more probably the suppressed HSD11B1 functioning (respecting the primarily local effects of HSD11B2 [62]).

Of the enzymes involved in or likely to influence cortisol synthesis, only the molar ratios related to CYP17A1 functioning in the hydroxylase step show elevated values in the Δ5 pathway, whereas the functioning of other enzymes is impaired in patients.

Most probably, the increased conversion of PREG to 17-OH-PREG, together with the decreased conversion of cortisol to cortisone, compensates for the aforementioned metabolic barriers, but this compensation appears to be insufficient in stressful situations in patients where blunted cortisol response and altered diurnal cortisol profile has been reported [20,21]. Again, it should be noted that the activities of the relevant enzymes were not measured directly, but changes in steroid levels and their molar ratios in patients were interpreted in relation to these enzymes.

**Figure 15:**
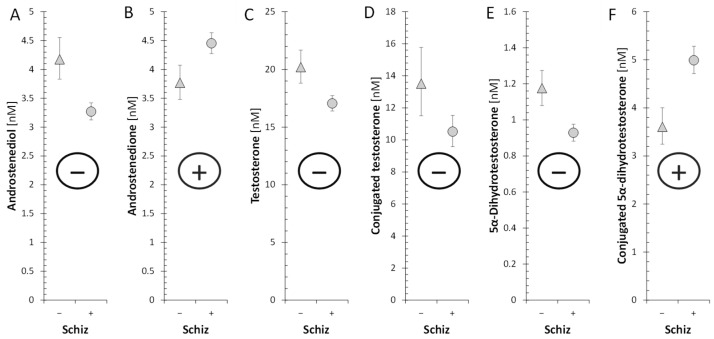
ANOVA model for evaluation of the effects of schizophrenia (Schiz), Age, and Stage of the experiment including Schiz × Stage interaction. Schiz (only the effects of schizophrenia are shown) on androstenedione and active androgens; (**A**): F = 12.9, *p* < 0.001, η_p_^2^ = 0.170; (**B**): F = 7.3, *p* = 0.009, η_p_^2^ = 0.109; (**C**): F = 8.88, *p* = 0.004, η_p_^2^ = 0.127; (**D**): F = 3.6, *p* = 0.062, η_p_^2^ = 0.059; (**E**): F = 11.0, *p* = 0.002, η_p_^2^ = 0.155; (**F**): F = 15.1, *p* < 0.001, η_p_^2^ = 0.203; F—F-statistics, *p*—*p*-value, η_p_^2^—effect size; interpreting η_p_^2^ (Cohen): 0.01~small, 0.06~medium, >0.14~large). The triangles and circles with error bars represent re-transformed means with 95% confidence intervals for controls and patients, respectively; C—symbolizes conjugated steroids.

**Figure 16:**
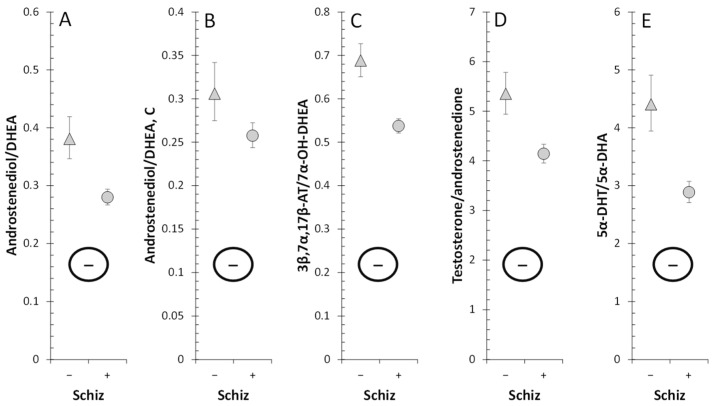
ANOVA model for evaluation of the effects of schizophrenia (Schiz), Age, and Stage of the experiment including Schiz × Stage interaction. Schiz (only the effects of schizophrenia are shown) on the molar ratios that may reflect a balance between reductive AKR1C3 and HSD17B3 on one side, and oxidative HSD17B2 on the other; (**A**): F = 17.3, *p* < 0.001, η_p_^2^ = 0.221; (**B**): F = 4.1, *p* = 0.047, η_p_^2^ = 0.0633; (**C**): F = 30.1, *p* < 0.001, η_p_^2^ = 0.338; (**D**): F = 15.1, *p* < 0.001, η_p_^2^ = 0.198; (**E**): F = 21.5, *p* < 0.001, η_p_^2^ = 0.254; F—F-statistics, *p*—*p*-value, η_p_^2^—effect size; interpreting η_p_^2^ (Cohen): 0.01~small, 0.06~medium, >0.14~large). The triangles and circles with error bars represent re-transformed means with 95% confidence intervals for controls and patients, respectively; C—symbolizes conjugated steroids. Symbols circled minus indicate attenuated conversion of Δ5 17β-hydroxy-androstanes to the corresponding 17-oxo-androstanes.

**Figure 17:**
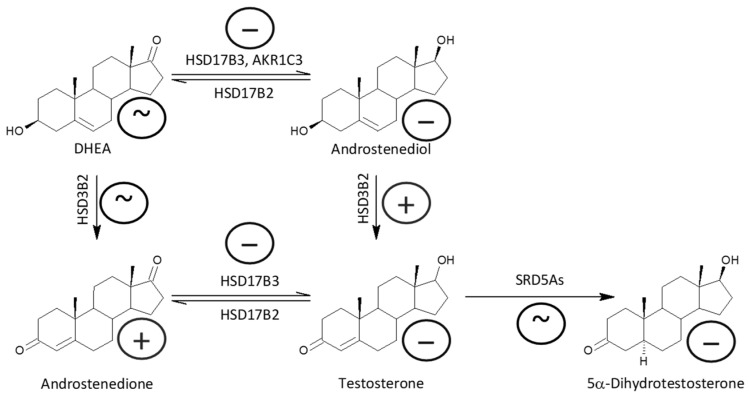
Simplified scheme of the alterations in the synthesis and metabolism of active androgens; the symbols +, ~, and −, represent higher, unaltered, and lower level of steroids or molar ratios, respectively.

Decreased testosterone and 5α-DHT levels in patients indicate impaired testicular function in patients. Therefore, we focused on the cause of this dysfunction at the level of steroidogenic enzymes involved in the synthesis of testosterone. While the levels of both unconjugated and conjugated T were lower in patients, androstenedione levels were higher. In addition, patients had lower levels of ADIOL (Figure 15). Moreover, the molar ratios, which may reflect the balance between HSD17B3 and AKR1C3 on the one hand, and HSD17B2 on the other, suggest a highly significant trend towards blunted conversion of 17-oxo androstanes to their 17β-hydroxy-counterparts in patients when compared to controls (Figure 16). The above results demonstrate suppressed HSD17B3 functioning in schizophrenic men, as this specifically testicular enzyme catalyzes the conversion of A to T in the Δ^4^ steroidogenic pathway and at the same time also the conversion of DHEA to ADIOL in the Δ^5^ steroidogenic pathway. ADIOL, like A, is also a precursor of T. In addition, levels of the most active androgen 5α-DHT were lower in the patients. However, levels of its conjugated form were higher, which may suggest that 5α-DHT conjugation may further contribute to the lack of active androgens in patients (Figure 17). Our previous study also reported elevated A, but found unaltered T levels in male patients as compared with corresponding controls [15].

**Figure 18:**
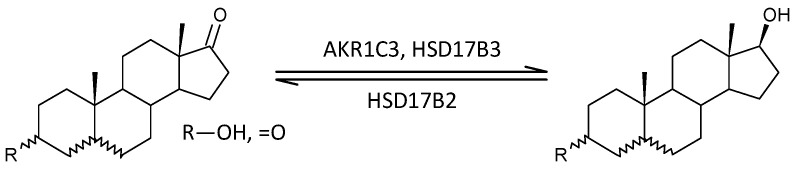
Scheme of the balance between type 1C2 aldoketoreductase (AKR1C3) and type 3 17β-hydroxysteroid dehydrogenase on one side, and type 2 and 6 17β-hydroxysteroid dehydrogenases (HSD17B2) on the other.

**Figure 19:**
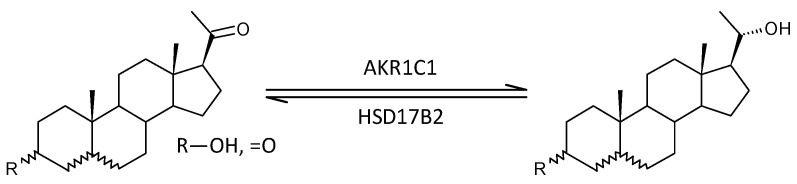
Scheme of the balance between type 1C1 aldoketoreductase (AKR1C1) and type 2 17β-hydroxysteroid dehydrogenase (HSD17B2).

**Figure 20:**
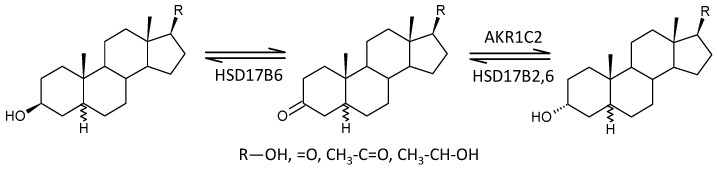
Scheme of the balance between type 1C2 aldoketoreductase (AKR1C2) on one side, and type 2 and 6 17β-hydroxysteroid dehydrogenases (HSD17B2 and HSD17B6) on the other.

On the one hand, our results may indicate a predominant shift from reductive enzymes, such as testicular HSD17B3 and possibly also extragonadal AKR1C3 (expressed in the *zona reticularis* of the adrenal gland and especially in adipocytes), to the oxidative enzyme HSD17B2 (Figure 18). However, given the unchanged molar ratios that may reflect the balance between AKR1C1 vs. HSD17B2 (Figure 19) and AKR1C2 vs. HSD17B2,6 (Figure 20), it is more likely that patients simply have impaired HSD17B3 and possibly also AKR1C3 function compared to controls. The blunted functioning of HSD17B3 is probably the main cause of T deficiency in patients, because the conversion of ADIOL to T is slightly but significantly increased, and the conversion of DHEA to A is not affected by the presence of schizophrenia, so these metabolic steps do not inhibit T synthesis. Further, because no consistent changes in SRD5A functioning were found in the patients, and there is no reason to expect an impediment in the conversion of T to 5α-DHT, the reduced 5α-DHT levels are therefore also indicative of a blockade of T synthesis from A in the patients; although, increased 5α-DHT conjugation may also contribute to the reduced 5α-DHT levels in the patients.

The question remains whether, in addition to the apparent block of A to T conversion in the testes, there is also a block of AKR1C3 functioning in extra-testicular tissues. Aside from specifically testicular HSD17B3, primarily extra-testicular AKR1C3 is a reductive enzyme that preferably converts 17-oxo steroids to their 17β-hydroxy counterparts; for example, it converts inactive A to the male sex hormone T, DHEA to ADIOL, and inactive estrone to the female sex hormone estradiol. AKR1C3 is highly expressed in immunocompetent cells, adipose tissue, intestine, smooth muscle, bronchial cells, colon, and liver, but its expression has also been detected in adrenal *zona reticularis* and in a variety of other tissues [66,67,68], http://biogps.org/#goto=genereport&id=8644, accessed on 26 June 2024) (see also reviews [69,70,71]).

Given that the majority of Δ^5^ androstanes are synthesized in the adrenal *zona reticularis* [72], it is likely that blunted conversion of DHEA to ADIOL, similar to the diminished conversion of 7α/β-hydroxy-DHEA to 5-androstene-3β,7α/β,17β-triols, could be primarily associated with reduced AKR1C3 functioning. This could also be relevant to the pathophysiology of schizophrenia because AKR1C3 also functions as prostaglandin (PG) F2α synthase, with PGF2α and its highly active metabolite 8-iso-PGF2α promoting oxidative stress and contributing to the inflammatory environment [73,74,75]. In addition, CNS inflammation and immune dysfunction are known to play a role in the pathogenesis of schizophrenia [76], and pro-inflammatory cytokines were elevated in the blood and cerebrospinal fluid of patients with schizophrenia [76]. Thus, the suppressed AKR1C3 function in patients suggested by our results could be involved in counterregulatory mechanisms that may alleviate the oxidative stress and inflammation that often accompany schizophrenia.

Our data generally show that steroidomic changes were not much affected by antipsychotic treatment, and if so, the changes were inconsistent. Thus, the steroidomic changes seem to be mainly related to the presence of schizophrenia. In addition, Ritsner et al. who monitored DHEA, DHEAS, P, A and T levels during antipsychotic treatment (at study entry and after 2, 4 and 8 weeks) found no significant changes during the experiment [31]. 

## 4. Limitations of this Study

Although this study includes a number of steroids and covers most of the steroid metabolic pathways, this study unfortunately does not include the determination of estrogens, whose role in the context of schizophrenia is best elucidated so far. The inclusion of estrogens would make it possible to evaluate any changes related to the functioning of aromatase (CYP19A1), which is an important steroidogenic enzyme in both sexes.

Another limitation is the absence of 11-deoxycortisol and 11-deoxy-corticosterone, the inclusion of which could lead to the evaluation of changes in CYP21A2 functioning, which is the only enzyme involved in the synthesis of cortisol for which data are missing in this study. Nevertheless, in the case of estimating the relationship of CYP11B1 functioning to schizophrenia in the present study, although the molar ratios of cortisol/11-deoxycortisol and corticosterone/11-deoxycorticosterone are missing, other suitable markers such as the molar ratios of 11β-hydroxy-androstanes to 11-deoxy-androstanes are available.

## 5. Materials and Methods

### 5.1. Subjects

The current research was performed as part of a long-term examination of individuals with first-episode schizophrenia (FES) conducted by the National Institute of Mental Health in the Czech Republic.

To be eligible for inclusion in the study, patients had to meet the following criteria: (1) be aged between 18 and 60 years, (2) receive a confirmed diagnosis of schizophrenia, acute polymorphic psychotic disorder, acute schizophrenia-like psychotic disorder, or schizoaffective disorder as determined by a psychiatrist using both the International Classification of Diseases-10 (ICD-10) criteria and the Mini International Neuropsychiatric Interview (M.I.N.I.), (3) experience early-stage psychosis, (4) have a duration of psychosis less than 24 months, and (5) be receiving or have previously received treatment with antipsychotic drugs at the time of evaluation.

Patients who suffered from organic mental disorders, intellectual disability with an IQ below 80, a history of seizures, traumatic brain injury with loss of consciousness, intracranial hemorrhage, severe neurological disorders, or substance addiction were excluded from this study.

This study was approved by the Ethical Committee of the National Institute of Mental Health, Klecany, Czech Republic (Approval number: 127/17), and all procedures involving human subjects were conducted following ethical standards set by national and institutional committees on human experimentation and the Helsinki Declaration of 1975, as updated in 2008. The authors guarantee that all research procedures were carried out with the utmost respect for the participants’ safety, well-being, and confidentiality.

A total of 51 adult male schizophrenics aged 27 (22, 34) years (shown as median with quartiles) and 16 HCs aged 28 (25, 32) years were evaluated. For the evaluation of the steroidome, the peripheral blood was withdrawn on fasting in the morning. Blood samples were centrifuged and stored at −20 °C until analyzed.

### 5.2. Steroid Analysis

Most steroids and deuterated standards were purchased from Steraloids (Newport, RI, USA). The deuterated standard D7 cortisone [2,2,4,6,6,12,12-D7] and trimethylchlorosilane (TMCS) for hydrolysis of steroids conjugates were from Sigma-Aldrich (St. Louis, USA). Sylon BTZ, methoxyamine hydrochloride and all other solvents and chemicals were from Merck (Darmstadt, Germany). All solvents were of HPLC grade.

Steroids and their polar conjugates were measured using our slightly modified GC-MS/MS method [77]. The modification consisted only in reducing the number of internal standards because we observed stability problems with [2,2,4,6,21-D8]-17α-hydroxyprogesterone and [2,2,4,6,17α,21-D9]-progesterone.

In brief, after addition of the mixed stock solution of internal standards such as [2,2,3,4,4,6-D6]-DHEA, [9,11,12,12-D4)-cortisol and [2,2,4,6,6,12,12-D7]-cortisone, into the serum sample and mixing (1 min), the unconjugated steroids were extracted from 1 mL of this mixture with diethyl-ether (3 mL). The diethyl-ether extract was dried in a block heater at 37 °C. The lipids in the dry residue of the diethyl-ether extract were separated by partitioning between a mixture of methanol with water 4:1 (1 mL) and pentane (1 mL). The pentane phase was discarded, and the polar phase was dried in a vacuum centrifuge at 60 °C (2 h). The dry residue from the polar phase was first dissolved in 100 μL of acetonitrile. The solution was transferred into a 1 mL conical vial and dried under the flow of nitrogen. The dry residue was derivatized first with a methoxyamine hydrochloride solution in pyridine (2%) (60 °C, 1 h) to convert the oxo-groups to methyloxime derivatives. After this first derivatization, the mixture was dried under a flow of nitrogen, and the dry residue was treated with the reagent Sylon BTZ (90 °C, 24 h). The Sylon BTZ is a mixture of N,O-bis(trimethylsilyl)acetamide (BSA) + trimethylchlorosilane (TMCS) + N-trimethylsilylimidazole (TMSI) (3:2:3). After this second derivatization step, the mixture was dried under the nitrogen flow (2 min). After administration of approximately 1 mg of ammonium bicarbonate, the residue was partitioned between isooctane (100 μL) and N,N-dimethylformamide (50 μL). Then, the volume of the vial was mixed (1 min) and centrifuged for 20 min at 3000 rpm. The lower, polar layer was aspirated with a Pasteur pipette and the upper, non-polar layer remained in the vial for GC-MS/MS analysis. From the upper layer, 2 μL was injected into the GC-MS/MS system.

Steroid conjugates remaining in the polar residue after diethyl ether extractions were analyzed as follows: The volume of 15 μL D6-DHEA sulfate solution (50 μg/mL) was mixed with this residue (1 min mixing). Then, 1 mL of methanol was added and mixed for an additional 1 min. After the centrifugation of the mixture (20 min at 3000 rpm), the upper layer was transferred to a clean 10 mL extraction tube, dried in the vacuum centrifuge at 37 °C (5 h), and the dry residues were chemically hydrolyzed according to Dehennin and Peres [78]. Briefly, 1 mL of 1 M TMCS was added to the dry residue of the upper layer, and after 1 min mixing the hydrolysis proceeded for 1 h at 55 °C. Then, 100 mg of sodium bicarbonate was added, and after short mixing the hydrolyzed samples were again dried in the vacuum centrifuge at 37 °C (5 h). The dried residues were reconstituted with 500 μL of chromatographic water, and then further processed in the same way as the free steroids.

The instrument used was a GCMS-TQ8040 system from Shimadzu (Kyoto, Japan) consisting of a gas chromatograph equipped with an automatic flow control, an AOC-20s autosampler, and a triple quadrupole detector with an adjustable electron voltage of 10–195 V. The analysis was conducted in multiple-reaction monitoring (MRM) mode. A capillary column with a medium polarity RESTEK Rtx-50 column (diameter 0.25 mm, length 15 m, film thickness 0.1 μm) was used for analyses. Electron-impact ionization with the electron voltage fixed at 60 V and emission current set to 151 μA was used for the measurements. The temperatures of the injection port, ion source, and interface were maintained at 220, 300, and 310 °C, respectively. Analyses were carried out in the splitless mode with a constant linear velocity of the carrier gas (He), which was maintained at 60 cm/s. The septum purge flow was set to 3 mL/min. The samples were injected using a high-pressure mode, which was applied at 200 kPa and maintained for 1 min. The detector voltage was set to 2.2 kV. The temperature program was as follows: 1 min delay at 80 °C, increase to 190 °C (40 °C/min), increase to 210 °C (6 °C/min), increase to 300 °C (20 °C/min), increase to 320 °C (40 °C/min), 4 min delay at 320 °C; initial pressure was 34 kPa, injector temperature was 220 °C, and analysis duration was 16.08 min.

The calibration was performed in a charcoal-stripped serum. The analytes were quantified using calibration curves based on known concentrations in the mixtures of analyzed standards with a constant level of ISs. We used a 9-point logarithmic calibration curve. The values were corrected for procedural losses according to yields of ISs. The amount of each steroid injected from the calibration samples into the GC-corresponded to amount of 10 ng, 2 ng, 500 pg, 125 pg, 31.2 pg, 7.81 pg, 1.95 pg, 488 fg, and 122 fg. The calibration curves were constructed by plotting the logarithm of response factor (analyte area/internal standard area) against the logarithm of concentration of the calibration (external) standard to cover the large concentration differences for circulating steroids in different physiological and pathophysiological situations and even more explicit contrasts between unconjugated steroids and their conjugated counterparts at an appropriate number of calibration points.

### 5.3. Statistical Analysis

**Figure 21:**
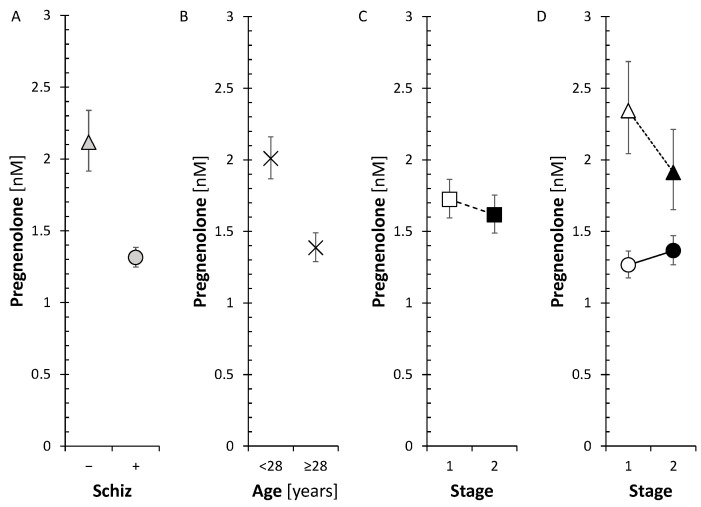
ANOVA model for evaluation of the effects of schizophrenia (Schiz), Age, and Stage of the experiment including Schiz × Stage interaction. Schiz: F = 35.6, *p* < 0.001, η_p_^2^ = 0.368; Age: F = 32, *p* < 0.001, η_p_^2^ = 0.344; Stage: F = 0.6, *p* = 0.428, η_p_^2^ = 0.0103; Schiz × Stage: F = 3, *p* = 0.088, η_p_^2^ = 0.0471; Subj (Schiz, Age): F = 4.2, *p* < 0.001, η_p_^2^ = 0.812; F—F-statistics, *p*—*p*-value, η_p_^2^—effect size; interpreting η_p_^2^ (Cohen): 0.01~small, 0.06~medium, >0.14~large). The triangles and circles with error bars represent re-transformed means with 95% confidence intervals for controls and patients, respectively. Panel A shows the effect of schizophrenia (patients vs. controls), panel B shows the effect of age (<28 years vs. ≥28 years), panel C shows the effect of stage (stage 2 vs. stage1), and panel D shows the schizophrenia × stage interaction.

In the first step, the power transformation parameters were found for each metric variable so that its distribution was as close as possible to the Gaussian distribution. The steroidomic data were evaluated using an ANOVA model followed by Bonferroni multiple comparisons. Due to age dependence for many of the steroidomic data, the ANOVA model included the factor Subject (explaining inter-individual variability), between-subject factors Schizophrenia (patients vs. controls) and Age (<28 vs. ≥28 years of age), the within-subject factor Stage (2—after one year vs. 1—initial stage), and the Schizophrenia × Stage interaction. Least significance multiple comparisons followed the ANOVA testing (*p* < 0.05) (for example see Figure 21). The statistical software Statgraphics Centurion v. XVIII from Statgraphics Technologies, Inc. (The Plains, VA, USA) was used for the above analyses.

The OPLS models were focused on the distinction between controls and patients. 

However, from the point of view of diagnosing schizophrenia based on steroidomic data, it was more appropriate to use OPLS models that examined the correlation of schizophrenia with multiple parameters simultaneously. These models allowed differentiation of patients from HCs. The OPLS model, which is a multivariate regression with dimensionality reduction, permits the evaluation of relationships between explanatory variables and a number of explanatory variables that may be highly correlated, which is also the case for steroids in metabolic pathways. The presence of the observed pathology is expressed in the OPLS model as the logarithm of the likelihood ratio (the ratio of the probability of the presence of pathology p to the probability of its absence (1-p)); i.e., the logarithm of the likelihood ratio is calculated, which then ranges from -infinity to +infinity. This approach ensures that the prediction of the probability of the presence of pathology is between 0 and 1 (after using a recurrent formula that converts the logarithm of the likelihood ratio to the probability of the presence of pathology).

The variability of the explaining and explained variables is separated into two independent components in the OPLS model. The former contains the variability in explaining variables that were shared with the probability of pathology (predictive component), while the orthogonal components express the variability shared in between highly correlated explaining variables (orthogonal components). The OPLS model identifies significant explanatory variables and their best linear combination to estimate the probability of the presence of pathology. After standardizing the variables, the OPLS model can be expressed as follows:

(1) $$X=T_{p}P_{p}^{T}+T_{0}P_{0}^{T}+E$$

(2) $$Y=T_{p}P_{p}^{T}+F$$

where ***X*** is the matrix with predictors and subjects; ***Y*** is the vector of dependent variable and subjects; ***T****_p_* is the vector of component scores from the single predictive component and subjects extracted from ***Y***; ***T***_0_ is the vector of component scores from the single orthogonal component and subjects extracted from ***X***; ***P****_p_* is the vector of component loadings for the predictive component extracted from ***Y***; ***P***_0_ is the vector of component loadings for the orthogonal component extracted from ***X*** and independent variables; and ***E*** and ***F*** are the error terms.

Significant predictors were selected using the variable importance statistics (VIPs). The statistical software SIMCA-P v.12.0 from Umetrics AB (Umeå, Sweden), which was used for the OPLS analysis, enabled finding the number of relevant components, the detection of multivariate non-homogeneities, and testing the multivariate normal distribution and homoscedasticity (constant variance).

The algorithm for obtaining the predictions was as follows:

- Transformation of the original data to obtain the values with symmetric distribution and constant variance;
- Checking the data homogeneity in predictors using Hotelling’s statistics and the eventual elimination of non-homogeneities;
- Testing the relevance of predictors using variable importance statistics and the elimination of irrelevant predictors;
- Calculating component loadings for individual variables to evaluate their correlations with the predictive component;
- Calculating regression coefficients for the multiple regression model to evaluate the mutual independence of predictors after comparison with the corresponding component loadings from the OPLS model;
- Calculating predicted values of the logarithm of the ratio of the probability of pathology presence to the probability of pathology absence (LLR);
- Calculating the probability of the pathology presence for individual subjects;
- Calculating the sensitivity and specificity of the prediction.

The ratio between significantly positive, missing, and significantly negative correlations with schizophrenia was evaluated using the Mann–Whitney test with correction for continuity.

## 6. Conclusions

In conclusion, the following are the main outcomes of the present study:

(1) Demonstrated the ability to effectively differentiate men with schizophrenia from controls, which helped to clarify the role of steroids in the pathophysiology of schizophrenia and suggested possibilities for their therapeutic use.
(2) Showed substantially altered adrenal and testicular steroidogenesis in the patients compared with controls.
(3) Showed an altered metabolic pathway from PREG/S to cortisol with several metabolic bottlenecks such as increased PREG sulfation and/or suppressed PREGS desulfation, lower pregnenolone levels, impaired conversion of 17-OH-PREG to 17-OH-P, and suppressed CYP11B1 function; however, two counterregulatory steps, increased conversion of PREG/S to 17-OH-PREG/S and decreased conversion of cortisol to cortisone, are likely to maintain unchanged basal cortisol levels but may not guarantee a sufficient cortisol response to stress.
(4) Indicated a trend towards higher 7α-, 7β- and 16α-hydroxylation whereby enzymes catalyzing these conversions may counteract the autoimmune complications and pro-inflammatory processes accompanying schizophrenia.
(5) Showed lower T levels at higher A levels, suggesting suppression of HSD17B3 functioning.

## Figures and Tables

**Table 1:** Discrimination between male schizophrenics and controls based on steroids as evaluated by an orthogonal predictions to latent structure (OPLS) model and multiple regression (MR).

				OPLS, Predictive Component							MR		
			Parameter	Variable importance (VIP)	t-statistics		Component loading	t-statistics	R ^a^		Regression coefficient	t-statistics	
Explaining Variables	Steroids		PREG	1.164	5.97	**	−0.169	−5.11	−0.576	**	−0.036	−4.51	**
			20α-Dihydro-PREG	1.012	4.05	**	−0.169	−6.75	−0.575	**	−0.032	−4.51	**
			17-OH-P	0.907	5.85	**	−0.173	−5.24	−0.588	**	−0.028	−4.40	**
			17-OH−20α-DHP	1.413	4.80	**	−0.185	−5.18	−0.629	**	−0.044	−3.45	**
			T	1.011	3.58	**	−0.157	−4.30	−0.535	**	−0.032	−3.85	**
			5α-DHT	0.745	3.13	**	−0.152	−4.59	−0.518	**	−0.023	−3.26	**
			3β,5α-THP	0.736	3.32	**	−0.135	−3.72	−0.460	**	−0.023	−3.23	**
			17-OH-ALLO	0.666	3.22	**	−0.150	−5.35	−0.509	**	−0.021	−3.05	**
			3β,5α,20α-PD	0.833	2.57	*	−0.138	−3.48	−0.470	**	−0.026	−2.72	*
			3α,5β,17,20α-PT	1.117	2.69	*	−0.153	−3.79	−0.521	**	−0.035	−2.16	*
			3α,5α-THA	0.895	2.23	*	−0.053	−1.13	−0.182		−0.028	−1.89	
			3β,5α,17β-AD	1.036	2.71	*	−0.105	−2.12	−0.358	*	−0.032	−2.26	*
	CYP17A1	hydroxylase + lyase	DHEA/PREG	1.51	29.63	**	0.239	15.17	0.816	**	0.047	9.96	**
			DHEA/PREGS	0.727	2.80	*	0.107	2.25	0.366	*	0.023	2.90	*
			DHEA/20α-Dihydro-PREG	1.137	5.55	**	0.206	10.40	0.702	**	0.035	8.17	**
			A/20α-DHP	0.777	2.80	*	0.147	3.76	0.500	**	0.024	2.72	*
			5α-DHA/5α,20α-THP	0.797	2.45	*	0.130	3.58	0.442	**	0.025	2.67	*
			3α,5α-THA, C/3α,5α,20α-PD, C	1.077	4.02	**	0.168	8.86	0.576	**	0.034	5.25	**
			3β,5α-THA/3β,5α-THP	0.925	3.44	**	0.164	5.77	0.542	**	0.029	3.69	**
			3β,5α-THA/3β,5α,20α-PD	0.895	2.74	*	0.154	5.52	0.511	**	0.028	3.23	**
		hydroxylase	17-OH-PREG/PREG	1.364	8.04	**	0.185	6.02	0.630	**	0.043	6.05	**
			17-OH-PREGS/PREGS	1.016	3.69	**	0.114	3.29	0.393	**	0.032	3.71	**
			16α-OH-PREG/PREG	1.073	6.83	**	0.165	4.29	0.562	**	0.033	4.92	**
		lyase	A/17-OH-P	1.287	4.85	**	0.218	10.14	0.742	**	0.040	3.77	**
			A/17-OH−20α-DHP	1.233	3.60	**	0.167	3.50	0.569	**	0.038	2.88	*
			3α,5α-THA, C/3α,5α,17,20α-PT, C	0.906	3.04	**	0.180	6.07	0.617	**	0.028	3.35	**
			3α,5β-THA, C/3α,5β,17,20α-PT, C	0.696	2.10	*	0.136	2.93	0.471	*	0.022	2.13	*
	HSD3Bs (HSD3B1,2)		17-OH-P/17-OH-PREG	1.214	4.74	**	−0.190	−4.98	−0.648	**	−0.038	−4.22	**
			17-OH-P/17-OH-PREGS	1.201	4.28	**	−0.171	−4.46	−0.587	**	−0.037	−4.31	**
	SULT2A1/STS		PREG, C/U	0.864	3.46	**	0.140	3.94	0.482	**	0.027	3.54	**
			5α-DHT, C/U	0.959	3.88	**	0.138	4.25	0.473	**	0.030	2.86	*
			3α,5α-THA, C/U	1.016	3.07	**	0.157	4.06	0.536	**	0.032	3.36	**
			3β,5α,17β-AD, C/U	0.826	2.81	*	0.159	4.62	0.547	**	0.026	2.48	*
	CYP11B1		11β-OH−3α,5α-THA, C/3α,5α-THA, C	0.832	2.87	*	−0.096	−2.85	−0.330	*	−0.026	−3.15	**
			11β-OH−3β,5α-THA/3β,5α-THA	1.109	3.88	**	−0.111	−2.69	−0.368	*	−0.035	−3.51	**
			11β-OH−3β,5α-THA, C/3β,5α-THA, C	0.94	3.49	**	−0.142	−5.84	−0.492	**	−0.029	−3.33	**
	SRD5As		(5α-DHT + 3α,5α,17β-AD + 3β,5α,17β-AD)/T, U + C	0.879	3.50	**	0.124	2.57	0.426	*	0.027	3.38	**
	AKR1C2/HSD17B2,6		3α,5β-THP, C/3β,5β-THP, C	0.741	2.80	*	−0.088	−2.95	−0.303	*	−0.023	−2.36	*
			3α,5α-THA, C/3β,5α-THA, C	0.646	2.75	*	0.100	4.99	0.343	**	0.020	3.01	**
	AKR1C3/HSD17B2		ADIOL/DHEA	0.963	3.12	**	−0.155	−4.42	−0.527	**	−0.030	−3.04	**
			T/A	0.964	5.63	**	−0.155	−6.36	−0.526	**	−0.030	−5.78	**
			5α-DHT/5α-DHA	1.105	4.66	**	−0.173	−7.15	−0.587	**	−0.034	−4.67	**
			3β,5α,17β-AD/3β,5α-THA	0.907	3.36	**	−0.175	−6.49	−0.581	**	−0.028	−3.39	**
Explained Variable			Male schizophrenics, LLR ^b^ (vs. controls)				1.000	17.71	0.695	**			
			Explained variability = 48.3% (43.4% after cross-validation), sensitivity = 0.923 (0.851–0.996), specificity = 0.917 (0.76–1), shown as means with 95% CI.										

^a^ R…Component loadings expressed as correlation coefficients with predictive component, * *p* < 0.05, ** *p* < 0.0, ^b^ —logarithm of likelihood ratio (probability of belonging to the selected group/probability of not belonging to the selected group). 17-OH-P—17-hydroxyprogesterone; 17-OH-PREG—17-hydroxypregnenolone; 17,20α-dihydroxy-4-pregnen-3-one—17-OH-20α-DHP; 5α-DHT—5α-dihydrotestosterone; DHEA—DHEA; 20α-DHP—20α-dihydroprogesterone; 5α-DHA—5α-androstane-3,17-dione; 5α,20α-THP—5α,20α-tetrahydroprogesterone; 3α,5α-THA—androsterone; 3α/β,5α/β,20α-PD—5α/β-pregnane-3α/β,20α-diols; 3α,5β-THP—pregnanolone; 3β,5α-THA—isopregnanolone; 3β,5β-THP—epipregnanolone; 3α/β,5α/β,17,20α-PT—5α/β-pregnane-3α/β,17,20α-triols; C/U—conjugated/unconjugated steroid; 3α/β,5α/β,17β-AD—5α/β,-androstane-3α/β,17β-diols; 11β-OH-3α,5α-THA—11β-hydroxyandrosterone; 11β-OH-3β,5α-THA—11β-hydroxyepiandrosterone.

**Table 2:** Differences between steroid levels in controls and patients found by an ANOVA model (factor Schizophrenia) including multiple comparisons in Stage 1 and indication of significant variables from an OPLS model for Stage 1 (symbols ↑ and ↓ represent positive and correlation with schizophrenia, respectively); * *p* < 0.05, ** *p* < 0.05, *** *p* < 0.001.

Steroids	Schizophrenia	Controls	ANOVA		MC, Stage 1	OPLS, Stage 1
Δ^5^ Pregnanes (C21 steroids)						
Pregnenolone [nM]	1.31 (1.25, 1.39)	2.12 (1.92, 2.34)	↓	***	↓	↓
Pregnenolone sulfate [nM]	397 (378, 417)	430 (394, 469)				
17-Hydroxypregnenolone [nM]	13.1 (12.3, 14)	12 (10.6, 13.6)				
17-Hydroxypregnenolone sulfate [nM]	32.3 (31, 33.7)	32 (29.6, 34.5)			↑	
16α-Hydroxypregnenolone [nM]	0.652 (0.629, 0.676)	0.723 (0.676, 0.772)				
20α-Dihydropregnenolone [nM]	2.69 (2.6, 2.78)	3.71 (3.49, 3.94)	↓	***	↓	↓
20α-Dihydropregnenolone sulfate [μM]	1.58 (1.5, 1.66)	1.92 (1.74, 2.12)	↓	*		
Δ^5^ Androstanes (C19 steroids)						
Dehydroepiandrosterone (DHEA) [nM]	11.9 (11.4, 12.4)	11.6 (10.7, 12.6)				
DHEA sulfate [μM]	7.51 (7.19, 7.84)	7.2 (6.67, 7.77)				
7α-Hydroxy-DHEA [nM]	1.33 (1.28, 1.39)	1.42 (1.31, 1.53)				
7-oxo-DHEA [nM]	0.381 (0.336, 0.432)	0.359 (0.279, 0.459)				
7β-Hydroxy-DHEA [nM]	0.693 (0.675, 0.712)	0.682 (0.647, 0.717)				
Androstenediol [nM]	3.27 (3.12, 3.42)	4.18 (3.83, 4.55)	↓	***	↓	
Androstenediol sulfate [μM]	2.21 (2.06, 2.37)	2.2 (1.93, 2.51)				
5-Androstene-3β,7α,17β-triol [nM]	0.698 (0.667, 0.73)	0.784 (0.718, 0.854)				
5-Androstene-3β,7β,17β-triol [nM]	0.349 (0.331, 0.368)	0.379 (0.342, 0.42)				
5-Androstene-3β,16α,17β-triol [pM]	187 (173, 201)	143 (123, 166)	↑	*		
5-Androstene-3β,16α,17β-triol sulfate [nM]	148 (137, 160)	131 (114, 150)				
Δ^4^ Pregnanes (C21 steroids)						
Progesterone [pM]	101 (83.5, 122)	152 (105, 228)				
17-Hydroxyprogesterone [nM]	3.25 (3.1, 3.41)	4.43 (4.03, 4.88)	↓	***	↓	↓
17,20α-Dihydroxy-4-pregnen-3-one [nM]	1.55 (1.47, 1.63)	2.52 (2.27, 2.79)	↓	***	↓	↓
Conjugated 17,20α-dihydroxy-4-pregnen-3-one [nM]	9.68 (9.2, 10.2)	11.9 (10.8, 13.1)	↓	*		
16α-Hydroxypogesterone [nM]	0.733 (0.526, 0.679)	0.824 (0.403, 0.594)				
20α-Dihydroprogesterone [pM]	162 (151, 174)	213 (188, 241)	↓	*	↓	↓
Conjugated 20α-dihydroprogesterone [nM]	1.44 (1.35, 1.54)	1.28 (1.13, 1.44)				
Δ^4^ Androstanes (C19 steroids)						
Androstenedione [nM]	4.45 (4.28, 4.64)	3.77 (3.48, 4.07)	↑	**		
Testosterone [nM]	17 (16.4, 17.7)	20.2 (18.8, 21.7)	↓	**	↓	↓
Conjugated testosterone [nM]	10.5 (9.57, 11.5)	13.5 (11.5, 15.8)				↓
5α Pregnanes (C21 steroids)						
5α-Dihydroprogesterone [pM]	33.6 (29.9, 37.7)	37.4 (30, 46.5)				
Allopregnanolone (3α,5α-THP) [pM]	107 (98.3, 117)	160 (137, 186)	↓	**	↓	
Allopregnanolone (3α,5α-THP) sulfate [nM]	6.46 (6.12, 6.82)	7.74 (7, 8.54)	↓	*		
Isopregnanolone (3β,5α-THP) [pM]	76.9 (76.6, 102)	128 (135, 212)	↓	**	↓	↓
Isopregnanolone (3β,5α-THP) sulfate [nM]	18.4 (17.7, 19.2)	20 (18.5, 21.6)				
17-Hydroxyallopregnanolone [pM]	23.4 (21.1, 25.9)	39.9 (33, 47.9)	↓	**	↓	↓
17-Hydroxyallopregnanolone sulfate [nM]	4.54 (4.31, 4.77)	5.21 (4.75, 5.68)				
5α,20α-Tetrahydroprogesterone [pM]	91.9 (86.1, 97.9)	109 (97.2, 122)			↓	
Conjugated 5α,20α-tetrahydroprogesterone [pM]	122 (109, 136)	78.9 (67.5, 93.2)	↑	**		
5α-Pregnane-3α,20α-diol [pM]	214 (196, 234)	217 (183, 256)				
Conjugated 5α-pregnane-3α,20α-diol [nM]	28.8 (27.3, 30.4)	38.8 (35.3, 42.6)	↓	***		
5α-Pregnane-3β,20α-diol [nM]	1.21 (1.03, 1.38)	1.94 (1.53, 2.28)	↓	*	↓	↓
Conjugated 5α-pregnane-3β,20α-diol [μM]	2.66 (2.52, 2.81)	3.58 (3.26, 3.92)	↓	***	↓	↓
5α-Pregnane-3α,17,20α-triol [pM]	277 (259, 295)	409 (367, 454)	↓	***	↓	↓
Conjugated 5α-pregnane-3α,17,20α-triol [nM]	57.2 (52.1, 62.7)	52.5 (44, 62.3)				
5α-Pregnane-3β,17,20α-triol [pM]	255 (232, 281)	361 (300, 433)	↓	*		
Conjugated 5α-pregnane-3β,17,20α-triol [nM]	5.12 (4.5, 5.83)	7.19 (5.71, 9.09)				
5β Pregnanes (C21 steroids)						
Pregnanolone (THP-3α,5;) [pM]	10.5 (9.21, 12.1)	13.6 (10.5, 17.5)				
Pregnanolone (3α,5β-THP) sulfate [nM]	27.8 (26.6, 29.1)	32.4 (29.8, 35.3)	↓	*		
Epipregnanolone (3β,5β-THP) sulfate [nM]	3.58 (2.5, 2.89)	3.75 (2.38, 2.97)				
17-Hydroxypregnanolone [pM]	40.8 (36.1, 45.9)	65.7 (53.2, 80.8)	↓	**	↓	
Conjugated 17-hydroxypregnanolone [nM]	16.5 (15.7, 17.3)	19.4 (17.9, 21.1)	↓	*	↓	
Conjugated 5β,20α-tetrahydroprogesterone [pM]	178 (167, 189)	249 (220, 281)	↓	***	↓	
5β-Pregnane-3α,20α-diol [pM]	97.1 (88.5, 106)	96 (79.8, 115)				
Conjugated 5β-pregnane-3α,20α-diol [nM]	13.5 (12.7, 14.3)	15.7 (14, 17.5)				
5β-Pregnane-3β,20α-diol [pM]	88.1 (74.6, 104)	68.1 (53.3, 87.5)				
Conjugated 5β-pregnane-3β,20α-diol [nM]	10.6 (14.2, 16.9)	10.9 (15.9, 21.1)				
5β-Pregnane-3α,17,20α-triol [nM]	1.8 (1.7, 1.91)	2.69 (2.45, 2.94)	↓	***	↓	
Conjugated 5β-pregnane-3α,17,20α-triol [nM]	128 (121, 136)	183 (163, 205)	↓	***	↓	
5α Androstanes (C19 steroids)						
5α-Androstane-3,17-dione [pM]	301 (286, 316)	249 (226, 275)	↑	*	↑	
Androsterone (THA-3α,5α) [nM]	1 (0.962, 1.04)	1.18 (1.09, 1.27)	↓	**	↓	
Androsterone (THA-3α,5α) sulfate [μM]	4.15 (3.94, 4.36)	3.24 (2.93, 3.58)	↑	**	↑	↑
Epiandrosterone (THA-3β,5α) [pM]	419 (400, 440)	387 (351, 426)				
Epiandrosterone (THA-3β,5α) sulfate [μM]	0.85 (0.807, 0.894)	0.73 (0.663, 0.802)	↑	*	↑	
5α-Dihydrotestosterone [nM]	0.929 (0.882, 0.976)	1.18 (1.08, 1.27)	↓	**	↓	↓
Conjugated 5α-dihydrotestosterone [nM]	4.99 (4.71, 5.28)	3.6 (3.24, 4)	↑	***	↑	
5α-Androstane-3α,17β-diol [pM]	388 (367, 410)	373 (331, 416)				
Conjugated 5α-androstane-3α,17β-diol [nM]	199 (191, 208)	195 (180, 212)				
5α-Androstane-3β,17β-diol [pM]	85.7 (79.5, 92.3)	128 (111, 147)	↓	***	↓	↓
Conjugated 5α-androstane-3β,17β-diol [nM]	190 (179, 202)	170 (153, 190)				
5β Androstanes (C19 steroids)						
Etiocholanolone (THA-3α,5β) [pM]	166 (159, 174)	157 (144, 171)				
Etiocholanolone (THA-3α,5β) sulfate [nM]	121 (115, 126)	105 (96, 114)	↑	*		
Epietiocholanolone (THA-3α,5β) sulfate [nM]	35.3 (32.7, 38.2)	36.1 (31.2, 41.7)				
5β-Androstane-3α,17β-diol [pM]	5.85 (5.18, 6.61)	4.91 (3.91, 6.18)				
Conjugated 5β-androstane-3α,17β-diol [nM]	15.9 (15.2, 16.7)	18.7 (17.2, 20.4)	↓	*		
Conjugated 5β-androstane-3β,17β-diol [nM]	0.61 (0.416, 0.517)	0.642 (0.433, 0.599)				
Glucocorticoids (Δ^4^ C21 steroids) and 11β-hydroxy-androstanes (Δ^4^, 5α/β- C19 steroids)						
Cortisol [nM]	378 (367, 389)	388 (368, 408)				
Cortisone [nM]	108 (103, 112)	130 (121, 140)	↓	**	↓	
Corticosterone [nM]	18.4 (17.1, 19.7)	17.2 (14.9, 19.6)			↑	
21-Deoxycortisol [pM]	39.1 (33.9, 45.3)	61.4 (46.6, 81.7)	↓	*		
11β-Hydroxyandrostenedione [nM]	39.6 (37.4, 41.8)	44 (39.7, 48.6)				
11β-Hydroxyandrosterone [nM]	2.21 (2.05, 2.37)	3.48 (3.06, 3.95)	↓	***	↓	↓
11β-Hydroxyandrosterone sulfate [nM]	44.5 (42.6, 46.5)	52.1 (48, 56.4)	↓	*		
11β-Hydroxyepiandrosterone [pM]	95.1 (87.7, 103)	142 (124, 162)	↓	***	↓	↓
11β-Hydroxyepiandrosterone sulfate [nM]	1.01 (0.939, 1.09)	1.57 (1.37, 1.81)	↓	***	↓	
11β-Hydroxyetiocholanolone [nM]	1.33 (1.24, 1.41)	1.56 (1.39, 1.75)			↓	
11β-Hydroxyetiocholanolone sulfate [nM]	8.37 (7.8, 8.98)	12.4 (11, 14)	↓	***	↓	

**Table 3:** Differences between steroid molar ratios that may reflect the functioning of steroidogenic enzymes and the balance between them in controls and patients found by an ANOVA model (factor Schizophrenia) including multiple comparisons in Stage 1 and indication of significant variables from an OPLS model for Stage 1 (symbols ↑ and ↓ represent positive and correlation with schizophrenia, respectively); * *p* < 0.05, ** *p* < 0.05, *** *p* < 0.001; ADIOL—androstenediol, ADIOLS—androstenediol sulfate, DHP—dihydroprogesterone, THP—tetrahydroprogesterone, PD—pregnanediol, PT—pregnanetriol, C...conjugated steroids, C+U—unconjugated steroids + conjugated steroids, A—androstenedione, THA—tetrahydroandrostenedione, T—testosterone, DHT—dihydrotestosterone, DHA5α—5α-androstane-3,17-dione, AD—androstanediol; activities of the relevant enzymes are not measured directly, but instead, the molar ratios of steroids are used, from which changes in steroidogenesis enzyme functioning or the balance between them can be qualitatively estimated in men with schizophrenia compared to controls.

Molar Ratios	Schizophrenia	Controls	ANOVA		MC, Stage 1	OPLS, Stage 1
C17-Hydroxylase, C17,20-lyase (CYP17A1), hydroxylase + lyase						
DHEA/PREG	5.49 (5.12, 5.88)	8.79 (8.49, 9.09)	↑	***	↑	↑
DHEA/PREG, C	16.2 (15.3, 17.2)	19.1 (18.4, 19.7)	↑	**	↑	↑
DHEA/20α-dihydro-PREG	3.01 (2.75, 3.29)	4.36 (4.14, 4.59)	↑	***	↑	↑
DHEA/20α-dihydro-PREG, C	3.23 (3.02, 3.46)	4.46 (4.29, 4.65)	↑	***	↑	↑
A/P	25.7 (18.2, 35.8)	40.1 (33.7, 47.5)				
A/20α-DHP	18.6 (17, 20.3)	27.9 (26.3, 29.5)	↑	***	↑	
5α-DHA/5α-DHP	7.38 (5.91, 9.23)	8.66 (7.69, 9.76)				
5α-DHA/5α,20α-THP	2.44 (2.21, 2.71)	3.48 (3.27, 3.71)	↑	***	↑	↑
3α,5α-THA/ALLO	6.81 (6.14, 7.58)	9.87 (9.31, 10.5)	↑	***	↑	
3α,5α-THA/3α,5α,20α-PD	5 (4.28, 5.88)	4.65 (4.27, 5.07)				
3α,5α-THA/ALLO, C	396 (368, 427)	633 (608, 660)	↑	***	↑	
3α,5α-THA/3α,5α,20α-PD, C	77.4 (71.8, 83.4)	130 (125, 136)	↑	***	↑	↑
3β,5α-THA/3β,5α-THP	3.27 (2.98, 3.58)	5.94 (5.62, 6.3)	↑	***	↑	↑
3β,5α-THA/3β,5α,20α-PD	0.236 (0.203, 0.275)	0.349 (0.32, 0.38)	↑	**	↑	↑
3β,5α-THA/3β,5α-THP, C	35.7 (33, 38.5)	44.7 (42.9, 46.5)	↑	***	↑	
3β,5α-THA/3β,5α,20α-PD, C	0.22 (0.2, 0.242)	0.326 (0.311, 0.342)	↑	***	↑	↑
3α,5β-THA/3α,5β-THP	11.7 (9.24, 14.9)	15.2 (13.3, 17.3)				
3α,5β-THA/3α,5β,20α-PD	1.66 (1.47, 1.89)	1.8 (1.68, 1.92)				
3α,5β-THA/3α,5β-THP, C	3.21 (3.02, 3.41)	4.26 (4.12, 4.41)	↑	***	↑	
3α,5β-THA/3α,5β,20α-PD, C	6.31 (5.83, 6.83)	8.33 (7.97, 8.71)	↑	***	↑	
3β,5β-THA/3β,5β-THP, C	9.82 (8.77, 11)	10.2 (9.56, 10.8)				
3β,5β-THA/3α,5β,20α-PD, C	3.56 (3.18, 3.98)	3.62 (3.4, 3.85)				
11β-OH-A/corticosterone	2.56 (2.3, 2.85)	2.32 (2.19, 2.46)			↓	
C17-Hydroxylase, C17,20-lyase (CYP17A1), hydroxylase						
10^3^·17-OH-PREG/PREG	66 (60.5, 71.8)	88.1 (84.5, 91.7)	↑	***	↑	↑
10^3^·17-OH-PREG/PREGS	5.96 (5.5, 6.46)	9.26 (8.85, 9.69)	↑	***	↑	↑
17-OH-P/P	31.2 (22.1, 43.4)	32.9 (27.5, 39.3)				
17-OH-20α-DHP/20α-DHP	31.2 (22.1, 43.4)	32.9 (27.5, 39.3)				
17-OH-20α-DHP/20α-DHP, C	10.1 (8.91, 11.4)	6.31 (5.94, 6.72)	↓	***	↓	↓
17-OH-ALLO/ALLO	0.27 (0.222, 0.331)	0.22 (0.199, 0.243)				
17-OH-ALLO/ALLO, C	0.735 (0.683, 0.79)	0.728 (0.7, 0.757)				
17-OH-3α,5β-THP/3α,5β-THP	3.57 (2.68, 4.75)	3.45 (2.96, 4.03)				
17-OH-3α,5β-THP/3α,5β-THP, C	0.56 (0.519, 0.603)	0.595 (0.572, 0.619)				
3α,5α,17,20α-PT/3α,5α,20α-PD	2.11 (1.75, 2.53)	1.58 (1.42, 1.76)				↓
3α,5α,17,20α-PT/3α,5α,20α-PD, C	1.2 (1.03, 1.41)	1.78 (1.63, 1.94)	↑	**	↑	
10^3^·3β,5α,17,20α-PT/3β,5α,20α-PD	192 (154, 238)	230 (205, 258)				
103·3β,5α,17,20α-PT/3β,5α,20α-PD, C	2.32 (1.78, 3.05)	2.11 (1.82, 2.45)				
3α,5β,17,20α-PT/3α,5β,20α-PD	25.7 (22, 30)	18.9 (17.5, 20.4)	↓	*	↓	↓
3α,5β,17,20α-PT/3α,5β,20α-PD, C	10.1 (8.74, 11.7)	8.97 (8.29, 9.7)				
Cortisol/corticosterone	23 (21, 25.2)	20.3 (19.3, 21.3)			↓	↓
C17-Hydroxylase, C17,20-lyase (CYP17A1), lyase						
DHEA/17-OH-PREG	0.933 (0.864, 1.01)	0.927 (0.888, 0.967)				
DHEA/17-OH-PREGS	227 (205, 252)	224 (212, 237)				
A/17-OH-P	0.856 (0.811, 0.904)	1.26 (1.22, 1.3)	↑	***	↑	↑
A/17-OH-20α-DHP	1.59 (1.43, 1.75)	2.8 (2.66, 2.93)	↑	***	↑	↑
3α,5α-THA/17-OH-ALLO	27.1 (23.1, 32.2)	41.4 (37.6, 45.6)	↑	**		
10^−3^·3α,5α-THA/17-OH-ALLO, C	0.532 (0.483, 0.586)	0.909 (0.862, 0.96)	↑	***	↑	↑
3α,5β-THA/17-OH-3α,5β-THP	3.13 (2.54, 3.89)	4.28 (3.8, 4.83)				↑
3α,5β-THA/17-OH-3α,5β-THP, C	5.24 (4.89, 5.62)	7.01 (6.74, 7.29)	↑	***	↑	↑
3α,5α-THA/3α,5α,17,20α-PT	2.66 (2.42, 2.94)	3.54 (3.33, 3.76)	↑	**	↑	
3α,5α-THA/3α,5α,17,20α-PT, C	61.8 (51.8, 73.4)	78.2 (71.1, 85.9)			↑	
3β,5α-THA/3β,5α,17,20α-PT	1.06 (0.908, 1.24)	1.55 (1.42, 1.69)	↑	**	↑	↑
3β,5α-THA/3β,5α,17,20α-PT, C	102 (80.8, 130)	151 (132, 172)	↑	*		↑
10^3^·3α,5β-THA/3α,5β,17,20α-PT	62.9 (57.3, 69)	96.6 (92.2, 101)	↑	***	↑	↑
3α,5β-THA/3α,5β,17,20α-PT, C	0.563 (0.503, 0.629)	0.938 (0.884, 0.996)	↑	***	↑	
10^3^·11β-OH-A/cortisol	111 (103, 120)	109 (104, 113)				
10^−3^·11β-OH-A/21-deoxycortisol	0.687 (0.528, 0.892)	0.969 (0.843, 1.11)				
3β-Hydroxysteroid dehydrogenases (type 1 and 2) (HSD3B1,2)						
10^3^·P/PREG	82.7 (60.5, 116)	72.9 (61.9, 86.4)				
10^3^·P/PREGS	0.352 (0.252, 0.506)	0.261 (0.22, 0.311)				
10^3^·20α-DHP/20α-dihydro-PREG	57 (50.9, 63.5)	58 (54.6, 61.4)				
10^3^·20α-DHP/20α-dihydro-PREGS	100 (85.7, 118)	83.4 (76.9, 90.4)				
103·20α-DHP/20α-dihydro-PREGS	0.699 (0.624, 0.782)	0.916 (0.865, 0.971)	↑	**		
17-OH-P/17-OH-PREG	0.374 (0.346, 0.404)	0.263 (0.253, 0.274)	↓	***	↓	↓
10^3^·17-OH-P/17-OH-PREGS	155 (144, 167)	106 (101, 111)	↓	***	↓	↓
16α-OH-P/16α-OH-PREG	1.24 (1.18, 1.31)	1.14 (1.1, 1.17)				
A/DHEA	0.376 (0.348, 0.407)	0.361 (0.346, 0.376)				
10^3^·A/DHEA sulfate	0.54 (0.479, 0.609)	0.544 (0.509, 0.58)				
T/ADIOL	4.66 (4.43, 4.91)	5.08 (4.93, 5.23)	↑	*		
10^3^·T, C/ADIOLS	7.15 (5.95, 8.59)	4.15 (3.75, 4.58)	↓	***	↓	↓
Conjugated/unconjugated steroid ratio—Steroid sulfotransferase 2A1 (SULT2A1) vs. steroid sulfatase (STS)						
PREG, C/U	215 (196, 236)	288 (275, 301)	↑	***	↑	↑
20α-Dihydro-PREG, C/U	595 (529, 671)	636 (599, 676)				
17-OH-PREG, C/U	2.61 (2.38, 2.86)	2.61 (2.48, 2.74)				
DHEA, C/U	609 (551, 676)	642 (609, 678)				
ADIOL, C/U	598 (525, 680)	656 (613, 703)				
10^−3^·3β,16α,17β-AT, C/U	1 (0.854, 1.18)	0.841 (0.774, 0.915)				
20α-DHP, C/U	6.36 (5.42, 7.5)	9.65 (8.82, 10.6)	↑	**	↑	↑
17-OH-20α-DHP, C/U	4.76 (4.17, 5.47)	5.9 (5.47, 6.38)			↑	↑
T, C/U	0.619 (0.505, 0.759)	0.581 (0.522, 0.647)				
5α-DHT, C/U	3.59 (3.1, 4.15)	6.43 (5.99, 6.9)	↑	***	↑	↑
ALLO, C/U	46.7 (41.2, 53.3)	64.5 (60, 69.6)	↑	**	↑	
3β,5α-THP, C/U	185 (163, 210)	252 (235, 270)	↑	**		↑
10^−3^·3α,5β-THP, C/U	2.63 (1.97, 3.51)	2.79 (2.4, 3.23)				
17-OH-ALLO, C/U	114 (95.5, 136)	187 (169, 207)	↑	**		
17-OH-3α,5β-THP, C/U	317 (260, 390)	413 (369, 464)				
5α,20α-THP, C/U	0.811 (0.665, 1)	1.43 (1.26, 1.62)	↑	**	↑	
3α,5α,20α-PD, C/U	213 (179, 253)	174 (158, 192)				
10^−3^·3β,5α,20α-PD, C/U	1.86 (1.6, 2.19)	2.4 (2.19, 2.63)			↑	
3α,5β,20α-PD, C/U	180 (150, 215)	174 (159, 191)				
3β,5β,20α-PD, C/U	149 (111, 197)	134 (110, 162)			↑	
3α,5α,17,20α-PT, C/U	97.8 (81.9, 117)	173 (156, 192)	↑	***	↑	
3β,5α,17,20α-PT, C/U	20 (17.1, 23.4)	20.8 (19.1, 22.6)				
10^−3^·3α,5β,17,20α-PT, C/U	2.66 (2.33, 3.03)	4.29 (4.03, 4.57)	↑	***		
10^−3^·3α,5α-THA, C/U	2.66 (2.33, 3.03)	4.29 (4.03, 4.57)	↑	***	↑	↑
10^−3^·3β,5α-THA, C/U	1.9 (1.65, 2.18)	1.93 (1.81, 2.06)				
10^−3^·3α,5β-THA, C/U	0.68 (0.621, 0.744)	0.782 (0.746, 0.819)			↑	
10^−3^·3α,5α,17β-AD, C/U	0.5 (0.452, 0.555)	0.512 (0.486, 0.539)				
10^−3^·3β,5α,17β-AD, C/U	1.53 (1.25, 1.89)	2.35 (2.12, 2.61)	↑	*		↑
10^−3^·3α,5β,17β-AD, C/U	2.39 (1.86, 3.08)	2.66 (2.34, 3.03)				
11β-OH-3α,5α-THA, C/U	15 (13.5, 16.7)	20.4 (19.2, 21.6)	↑	**		↑
11β-OH-3β,5α-THA, C/U	10.1 (8.65, 11.8)	10.5 (9.67, 11.5)				
11β-OH-3β,5α-THA, C/U	8.2 (7.08, 9.44)	6.7 (6.16, 7.28)				
11β-Hydroxylase (CYP11B1)						
11β-OH-A/A	11.1 (10.2, 12.1)	8.96 (8.56, 9.38)	↓	**	↓	
11β-OH-3α,5α-THA/3α,5α-THA	3.03 (2.68, 3.4)	2.36 (2.2, 2.53)	↓	*	↓	↓
10^3^·11β-OH-3α,5α-THA/3α,5α-THA, C	16.4 (14.7, 18.3)	10.9 (10.3, 11.5)	↓	***	↓	↓
11β-OH-3β,5α-THA/3β,5α-THA	0.348 (0.303, 0.398)	0.234 (0.216, 0.254)	↓	**	↓	↓
10^3^·11β-OH-3β,5α-THA/3β,5α-THA, C	1.9 (1.67, 2.17)	1.17 (1.1, 1.25)	↓	***	↓	↓
11β-OH-3β,5α-THA/3α,5β-THA	10.1 (9.25, 11.1)	8.53 (8.11, 8.97)	↓	*	↓	
10^3^·11β-OH-3β,5α-THA/3α,5β-THA, C	94.8 (83.4, 108)	67.6 (63, 72.5)	↓	**	↓	↓
7α/β-,16α-Hydroxylases (CYP7B1, CYP3A4,7)						
10^3^·7α-OH-DHEA/DHEA	118 (110, 126)	114 (110, 118)				
10^3^·3β,7α,17β-AT/ADIOL	180 (168, 193)	210 (203, 218)	↑	**	↑	
10^3^·7β-OH-DHEA/DHEA	52 (47.8, 56.6)	58.4 (55.9, 61.1)				
10^3^·3β,7β,17β-AT/ADIOL	88.5 (82.1, 95.4)	110 (105, 114)	↑	***	↑	
10^3^·16α-OH-PREG/PREG	347 (322, 373)	448 (430, 467)	↑	***	↑	↑
10^3^·3β,16α,17β-AT/ADIOL	32.1 (27.8, 36.8)	54.5 (50.8, 58.4)	↑	***	↑	↑
10^3^·3β,16α,17β-AT/ADIOLS	54.8 (49.1, 61.4)	62.8 (58.9, 67)				
16α-OH-P/P	5.82 (4.27, 7.8)	7.73 (6.62, 9)				
11β-Hydroxysteroid dehydrogenase, type 1 (HSD11B1)						
7-oxo-DHEA/7α-OH-DHEA	0.285 (0.225, 0.355)	0.323 (0.287, 0.362)				
7β-OH-DHEA/7α-OH-DHEA	0.475 (0.448, 0.503)	0.533 (0.516, 0.549)	↑	*		
3β,7β,17β-AT/3β,7α,17β-AT	0.493 (0.47, 0.518)	0.523 (0.51, 0.538)				
Cortisol/cortisone	3.04 (2.83, 3.26)	3.32 (3.19, 3.44)				
5α-Reductases, type 1 and 2 (SRD5A1,2)						
(5α-DHP+3α/β,5α-THP)/P	1.76 (1.26, 2.43)	1.98 (1.66, 2.35)				
(3α/β,5α-THP, C)/P	169 (115, 243)	252 (209, 303)				
(5α-DHP+3α/β,5α-THP+3α/β,5α-THP, all C)/P	169 (115, 243)	252 (209, 303)				
(5α,20α-THP+3α/β,5α,20α-PD)/20α-DHP	10.1 (8.78, 11.7)	9.67 (8.95, 10.5)				
10^−3^·(5α,20α-THP+3α/β,5α,20α-PD)/20α-DHP, all C	2.44 (2.19, 2.71)	1.79 (1.7, 1.9)	↓	***	↓	
10^−3^·(5α,20α-THP+3α/β,5α,20α-PD)/20α-DHP, all C+U	1.94 (1.73, 2.16)	1.56 (1.47, 1.65)	↓	*		
10^3^·3α,5α,17-PD/17-OH-P	9.87 (8.22, 11.8)	6.64 (6.03, 7.31)	↓	**		
3α,5α,17-PD, C/17-OH-P	1.08 (0.973, 1.21)	1.31 (1.24, 1.39)	↑	*	↑	
3α,5α,17-PD, C+U/17-OH-P	1.07 (0.936, 1.21)	1.34 (1.25, 1.43)	↑	*	↑	
3α,5α,17,20α-PT/17-OH-20α-DHP	0.281 (0.244, 0.324)	0.322 (0.299, 0.346)				
3α,5α,17,20α-PT/17-OH-20α-DHP, all C	4.66 (3.95, 5.51)	6.53 (5.94, 7.19)	↑	*		
3α,5α,17,20α-PT/17-OH-20α-DHP, all C+U	3.82 (3.25, 4.49)	5.54 (5.06, 6.07)	↑	**		
(5α-DHA+3α/β,5α-THA)/A	0.437 (0.406, 0.47)	0.384 (0.37, 0.398)	↓	*	↓	
10^−3^·3α/β,5α-THA, C/A	0.971 (0.846, 1.11)	1.16 (1.08, 1.24)			↑	↑
10^−3^·(5α-DHA+3α/β,5α-THA, all C+U)/A	0.992 (0.863, 1.13)	1.15 (1.08, 1.23)				↑
10^−3^·(5α-DHT+3α/β,5α-AD)/T	84 (78.4, 90.1)	78.7 (75.8, 81.6)				
(5α-DHT+3α/β,5α-AD)/T, all C	31.1 (25.2, 38.4)	44.3 (39.5, 49.6)	↑	*		↑
(5α-DHT+3α/β,5α-AD)/T, all C+U	11.8 (10.3, 13.3)	14.6 (13.7, 15.6)	↑	*	↑	↑
10^3^·11β-OH-3α/β,5α-THA/11β-OH-A	82.7 (74.7, 91.1)	62.2 (58.5, 66)	↓	**	↓	
11β-OH-3α/β,5α-THA, C/11β-OH-A	1.17 (1.06, 1.3)	1.18 (1.12, 1.25)				
11β-OH-3α/β,5α-THA, C+U/11β-OH-A	1.27 (1.15, 1.4)	1.25 (1.19, 1.32)				
5β-Reductase (AKR1D1)						
10^3^·3α,5β-THP/P	76 (49.5, 115)	92.1 (73.7, 115)				
3α/β,5β-THP, C/P	230 (156, 333)	323 (267, 390)				
3α/β,5β-THP, C+U/P	230 (156, 333)	323 (267, 390)				
(3α/β,5β,20α-PD)/20α-DHP	0.834 (0.699, 1)	1.09 (0.963, 1.25)				
(5β,20α-THP+3α/β,5β,20α-PD)/20α-DHP, all C	19.8 (17.7, 22.2)	16.4 (15.5, 17.4)	↓	*		
(5β,20α-THP+3α/β,5β,20α-PD)/20α-DHP, all C+U	16.2 (14.5, 18.1)	16.4 (15.2, 17.6)			↑	
10^3^·3α,5β,17-PD/17-OH-P	11.9 (8.96, 15.6)	11.6 (9.93, 13.4)				
3α,5β,17-PD, C/17-OH-P	4.02 (3.65, 4.43)	4.94 (4.69, 5.2)	↑	*		
3α,5β,17-PD, C+U/17-OH-P	4.03 (3.66, 4.44)	4.96 (4.7, 5.22)	↑	*		
3α,5β,17,20α-PT/17-OH-20α-DHP	0.985 (0.89, 1.09)	1.05 (0.992, 1.1)				
3α,5β,17,20α-PT/17-OH-20α-DHP, all C	12.2 (10.8, 13.7)	12.8 (12.1, 13.6)				
3α,5β,17,20α-PT/17-OH-20α-DHP, all C+U	9.98 (8.91, 11.2)	11 (10.4, 11.7)				
10^3^·3α,5β-THA/A	40.5 (36.6, 44.8)	36.9 (34.9, 39)				
3α/β,5β-THA, C/A	37.3 (32.7, 42.6)	37.2 (34.7, 39.9)				
3α/β,5β-THA, C+U/A	37.3 (32.7, 42.6)	37.2 (34.7, 39.9)				
3α,5β,17β-AD/T	1.42 (1.2, 1.68)	1.66 (1.51, 1.83)				
3α/β,5β,17β-AD/T, C	1.42 (1.2, 1.68)	1.66 (1.51, 1.83)				
3α/β,5β,17β-AD/T, C+U	0.554 (0.499, 0.615)	0.561 (0.531, 0.592)				
10^3^·11β-OH-3α,5β-THA/11β-OH-A	38.2 (35.4, 41.1)	33.1 (31.7, 34.6)	↓	*	↓	
11β-OH-3α,5β-THA, C/11β-OH-A	0.269 (0.228, 0.317)	0.215 (0.196, 0.236)				
11β-OH-3α,5β-THA, C+U/11β-OH-A	0.314 (0.273, 0.363)	0.251 (0.233, 0.272)				
Aldoketoreductase 1C1 (AKR1C1) vs. 17β-hydroxysteroid dehydrogenase, type 2 (HSD17B2)						
20α-Dihydro-PREG/PREG	1.78 (1.62, 1.95)	1.88 (1.79, 1.98)				
20α-Dihydro-PREGS/PREGS	4.78 (4.59, 4.98)	4.07 (3.98, 4.15)	↓	***	↓	
20α-DHP/P	1.39 (1.01, 1.89)	1.5 (1.27, 1.76)				
20α-DHP, C/P	7.89 (5.41, 11.4)	13.5 (11.1, 16.4)			↑	
17-OH-20α-DHP/17-OH-P	0.523 (0.479, 0.574)	0.456 (0.437, 0.477)				
17-OH-20α-DHP, C/17-OH-P	2.69 (2.41, 3.01)	2.95 (2.77, 3.13)			↑	
5α,20α-THP/5α-DHP	2.65 (2.11, 3.33)	2.35 (2.07, 2.66)				
5α,20α-THP, C/5α-DHP	2.1 (1.61, 2.79)	3.33 (2.85, 3.91)	↑	*		
3α,5α,20α-PD/ALLO	1.16 (1.01, 1.33)	2.03 (1.87, 2.2)	↑	***	↑	
3α,5α,20α-PD, C/ALLO, C	5.27 (4.95, 5.61)	4.57 (4.43, 4.72)	↓	**	↓	
3β,5α,20α-PD/3β,5α-THP	13.6 (11.6, 16.1)	15.1 (13.9, 16.6)				
3β,5α,20α-PD, C/3β,5α-THP, C	173 (165, 182)	141 (137, 145)	↓	***	↓	
3α,5β,20α-PD/3α,5β-THP	7.42 (5.79, 9.57)	7.58 (6.64, 8.67)				
3α,5β,20α-PD, C/3α,5β-THP, C	0.488 (0.448, 0.532)	0.498 (0.475, 0.522)				
3β,5β,20α-PD, C/3α,5β-THP, C	2.84 (2.65, 3.05)	3 (2.89, 3.11)				
3α,5α,17,20α-PT/17-OH-ALLO	8.37 (7.26, 9.7)	11.3 (10.5, 12.3)	↑	*		
3α,5α,17,20α-PT, C/17-OH-ALLO, C	9.33 (8.01, 10.9)	11.3 (10.4, 12.3)				
3α,5β,17,20α-PT/17-OH-3α,5β-THP	42.5 (35.9, 50.6)	45.1 (41, 49.6)				
3α,5β,17,20α-PT, C/17-OH-3α,5β-THP, C	7.69 (6.95, 8.51)	7.68 (7.28, 8.11)				
Aldoketoreductase 1C2 (AKR1C2) vs. 17β-hydroxysteroid dehydrogenases, type 2 and 6 (HSD17B2,6)						
ALLO/3β,5α-THP	1.43 (1.25, 1.63)	1.63 (1.52, 1.74)				
ALLO, C/3β,5α-THP, C	0.383 (0.353, 0.415)	0.338 (0.323, 0.352)				
3α,5β-THP, C/3β,5β-THP, C	8.69 (8.19, 9.22)	7.83 (7.59, 8.09)	↓	*	↓	
1000·3α,5α,20α-PD/3β,5α,20α-PD	118 (99.5, 141)	162 (148, 179)	↑	*		
1000·3α,5α,20α-PD, C/3β,5α,20α-PD, C	11.2 (10.4, 12.1)	11 (10.5, 11.4)				
3α,5β,20α-PD, C/3β,5β,20α-PD, C	1.49 (1.39, 1.6)	1.36 (1.31, 1.41)				
3α,5α,17,20α-PT/3β,5α,17,20α-PT	1.05 (0.943, 1.17)	1.1 (1.04, 1.17)				
3α,5α,17,20α-PT, C/3β,5α,17,20α-PT, C	6.63 (5.22, 8.48)	9.39 (8.2, 10.8)				
3α,5α-THA/3β,5α-THA	6.63 (5.22, 8.48)	9.39 (8.2, 10.8)				
3α,5α-THA, C/3β,5α-THA, C	4.31 (4.05, 4.57)	4.76 (4.61, 4.92)	↑	*	↑	↑
3α,5β-THA/3β,5β-THA, C	3.1 (2.79, 3.42)	3.6 (3.41, 3.79)				
3α,5α,17β-AD, C/3β,5α,17β-AD, C	2.79 (2.39, 3.23)	4.62 (4.29, 4.98)	↑	***	↑	
3α,5α,17β-AD, C/3β,5α,17β-AD, C	1.27 (1.19, 1.36)	1.06 (1.02, 1.1)	↓	**		
3α,5β,17β-AD, C/3β,5β,17β-AD, C	32 (28.6, 35.9)	26.4 (24.9, 28)	↓	*	↓	
11β-OH-3α,5α-THA/11β-OH-3β,5α-THA	32 (28.6, 35.9)	26.4 (24.9, 28)	↓	*	↓	
11β-OH-3α,5α-THA, C11β-OH-3β,5α-THA, C	35.4 (31.5, 39.9)	41.9 (39.1, 44.8)				
ALLO/5α-DHP	3.9 (3.15, 4.81)	2.86 (2.54, 3.21)			↓	
ALLO, C/5α-DHP	191 (152, 241)	184 (163, 207)				
ALLO, C+U/5α-DHP	196 (156, 247)	188 (168, 212)				
3α,5α,20α-PD/5α,20α-THP	1.82 (1.54, 2.15)	2.15 (1.96, 2.37)				
3α,5α,20α-PD, C/5α,20α-THP, C	387 (325, 464)	221 (203, 241)	↓	***	↓	
3α,5α,20α-PD, C+U/5α,20α-THP, C+U	407 (353, 471)	361 (335, 389)				
3α,5β,20α-PD, C/5β,20α-THP, C	70 (62.9, 77.9)	76.4 (72, 81)			↑	
3α,5α-THA/5α-DHA	4.2 (3.78, 4.68)	3.26 (3.08, 3.45)	↓	**	↓	
10^−3^·3α,5α-THA, C/5α-DHA	12.7 (10.9, 14.6)	14.3 (13.3, 15.3)				↑
10^−3^·3α,5α-THA, C+U/5α-DHA	12.7 (10.9, 14.6)	14.3 (13.3, 15.3)				↑
17β-Hydroxysteroid dehydrogenase, type 3 (HSD17B3) + aldoketoreductase 1C3 (AKR1C3) vs. 17β-hydroxysteroid dehydrogenase, type 2 (HSD17B2)						
ADIOL/DHEA	0.381 (0.346, 0.419)	0.28 (0.267, 0.294)	↓	***	↓	↓
ADIOLS/DHEAS	0.306 (0.275, 0.342)	0.257 (0.244, 0.272)	↓	*		
3β,7α,17β-AT/7α-OH-DHEA	0.688 (0.651, 0.727)	0.537 (0.521, 0.554)	↓	***	↓	↓
3β,7β,17β-AT/7β-OH-DHEA	0.616 (0.571, 0.665)	0.549 (0.527, 0.573)				
T/A	5.35 (4.94, 5.78)	4.14 (3.96, 4.33)	↓	***	↓	↓
T, C/A	3.31 (2.7, 4.04)	2.28 (2.04, 2.56)	↓	*		
T, C+U/A	8.75 (7.78, 9.83)	6.96 (6.53, 7.42)	↓	*		
5α-DHT/5α-DHA	4.4 (3.94, 4.91)	2.89 (2.71, 3.07)	↓	***	↓	↓
5α-DHT, C/5α-DHA	14.3 (12.4, 16.6)	16.9 (15.7, 18.3)				
5α-DHT, C+U/5α-DHA	19 (16.6, 21.8)	20.4 (19, 22)				
10^3^·3α,5α,17β-AD/3α,5α-THA	352 (328, 376)	407 (394, 420)	↑	**		
10^3^·3α,5α,17β-AD, C/3α,5α-THA, C	61.8 (58, 66)	49.5 (47.9, 51.1)	↓	***	↓	↓
10^3^·3β,5α,17β-AD/3β,5α-THA	340 (302, 383)	225 (211, 240)	↓	***	↓	↓
10^3^·3β,5α,17β-AD/3β,5α-THA, C	239 (219, 261)	232 (221, 244)				
10^3^·3α,5β,17β-AD/3α,5β-THA, C	174 (163, 185)	134 (129, 139)	↓	***	↓	↓
10^3^·3β,5β,17β-AD/3β,5β-THA, C	14 (12.7, 15.4)	15.9 (15, 16.7)				

PREG—pregnenolone; DHEA—dehydroepiandrosterone; ADIOL—androstenediol; ADIOLS— androstenediol sulfate; C—conjugated steroids; 17-OH-—17-hydroxy-; A—androstenedione; P—progesterone; DHP—dihydroprogesterone; 5α-DHA—5α-androstane-3,17-dione; 3α,5α-THA—androsterone; 3β,5α-THA—epiandrosterone; 3α,5β-THA—etiocholanolone; 3β,5β-THA—epietiocholanolone; 17,20α-dihydroxy-4-pregnen-3-one—17-OH-20α-DHP; 5α-DHT—5α-dihydrotestosterone; THP—tetrahydroprogesterone; 3α/β,5α/β-20α-PD—5α/β-pregnane-3α/β,20α-diol; 3α/β,5α/β,17,20α-PT—5α/β-pregnane-3α/β,17,20α-triol; 11β-OH-—11β-hydroxy-; C/U—conjugated/unconjugated steroid; 3α/β,5α/β,17β-AD—5α/β,-androstane-3α/β,17β-diols; 11β-OH-3α,5α-THA—11β-hydroxyandrosterone; 11β-OH-3β,5α-THA—11β-hydroxyepiandrosterone, C+U conjugated + unconjugated steroids, all C—all steroids are conjugated, all C+U— all conjugated and unconjugated steroids are included.

**Table 4:** Significant Schizophrenia × Stage interactions, showing distinct changes between Stage 1 and Stage 2 for patients and controls for some steroids and steroid molar ratios. (Symbols ↑ and ↓ represent positive and correlation with schizophrenia, respectively; * *p* < 0.05, ** *p* < 0.05, *** *p* < 0.001; MC—LSD multiple comparisons.)

Steroids and Steroid Molar Ratios	Controls		Schizophrenia		ANOVA, Stage main factor		MC, Controls	MC, Schizophrenia	ANOVA, Schizophrenia × Stage
	Stage 1	Stage 2	Stage 1	Stage 2					
17-OH-PREGS [nM]	27.6 (24.8, 30.7)	37 (33.1, 41.3)	34.8 (32.7, 37)	30 (28.2, 31.9)			↑	↓	***
ADIOL [nM]	4.91 (4.35, 5.56)	3.56 (3.15, 4.04)	3.33 (3.12, 3.55)	3.21 (3.01, 3.42)	↓		↓		*
16α-OH-P [nM]	0.74 (0.625, 0.874)	0.915 (0.767, 1.09)	0.841 (0.769, 0.918)	0.637 (0.581, 0.698)				↓	*
3α,5α,20α-PD [pM]	248 (197, 308)	190 (146, 242)	174 (152, 198)	262 (232, 294)				↑	*
3β,5α,20α-PD [nM]	2.35 (1.98, 2.77)	1.58 (1.29, 1.92)	1.1 (0.975, 1.23)	1.34 (1.2, 1.49)			↓		**
3β,5β,20α-PD [pM]	105 (74, 150)	45 (31.5, 64.5)	67.7 (53.9, 85.3)	116 (90.8, 148)			↓	↑	**
3β,5β,20α-PD, C [nM]	10.1 (8.74, 11.7)	11.7 (10, 13.6)	11.8 (10.8, 12.8)	9.51 (8.75, 10.3)				↓	*
3α,5α-THA sulfate [μM]	2.88 (2.48, 3.31)	3.63 (3.15, 4.16)	4.36 (4.07, 4.67)	3.94 (3.66, 4.23)					*
Corticosterone [nM]	15.2 (12.4, 18.4)	19.3 (15.8, 23.2)	20.8 (18.8, 22.9)	16.1 (14.4, 17.9)				↓	*
11β-OH-3β,5α-THA [pM]	155 (129, 185)	130 (105, 158)	78.2 (69.2, 88)	114 (103, 127)				↑	*
DHEA/PREG	4.81 (4.37, 5.29)	6.23 (5.63, 6.89)	9.4 (8.95, 9.86)	8.22 (7.83, 8.62)			↑	↓	***
DHEA/20α-dihydro-PREG	2.65 (2.34, 2.99)	3.42 (3, 3.92)	4.65 (4.31, 5.02)	4.09 (3.81, 4.4)			↑		*
3α,5α-THA/3α,5α,20α-PD	4.66 (3.79, 5.82)	5.37 (4.27, 6.87)	5.93 (5.22, 6.78)	3.7 (3.3, 4.16)				↓	*
11β-OH-A/corticosterone	3.08 (2.66, 3.59)	2.13 (1.84, 2.49)	2.17 (2, 2.35)	2.48 (2.29, 2.7)			↓		**
17-OH-PREG/PREG	5.17 (4.63, 5.77)	6.88 (6.1, 7.76)	10.8 (10.2, 11.6)	7.94 (7.46, 8.46)			↑	↓	***
10^3^·17-OH-PREG/PREG, C	58.5 (51.1, 66.3)	74 (65.8, 82.8)	97.5 (92.3, 103)	79.1 (74.4, 83.9)				↓	**
Cortisol/corticosterone	25 (22, 28.4)	21.2 (18.6, 24.2)	17.6 (16.4, 18.8)	23.4 (21.8, 25.2)				↑	**
DHEA/17-OH-PREG, C	254 (220, 293)	204 (176, 236)	206 (190, 223)	244 (225, 264)				↑	*
A/17-OH-P	0.791 (0.734, 0.852)	0.927 (0.855, 1.01)	1.28 (1.22, 1.34)	1.24 (1.18, 1.29)			↑		*
10^−3^·3α,5α-THA/17-OH-ALLO, C	0.486 (0.424, 0.556)	0.583 (0.509, 0.669)	1 (0.929, 1.08)	0.825 (0.765, 0.889)				↓	*
17-OH-P/17-OH-PREG	0.446 (0.399, 0.5)	0.315 (0.283, 0.352)	0.258 (0.243, 0.273)	0.269 (0.253, 0.286)	↓	*	↓		**
10^3^·17-OH-P/17-OH-PREGS	209 (189, 231)	114 (101, 128)	106 (99.2, 113)	106 (99.6, 113)	↓	***	↓		***
16α-OH-P/16α-OH-PREG	1.1 (1.02, 1.19)	1.39 (1.29, 1.5)	1.19 (1.14, 1.24)	1.09 (1.04, 1.14)			↑	↓	***
17-OH-20α-DHP, C/U	4 (3.35, 4.8)	5.72 (4.66, 7.12)	6.34 (5.67, 7.11)	5.5 (4.97, 6.12)					*
ALLO, C/U	38.2 (32.6, 45.2)	57.8 (47.2, 71.6)	70.7 (63.4, 79)	59.1 (53.6, 65.4)			↑		**
3α,5α,20α-PD, C/U	168 (131, 214)	267 (207, 342)	219 (191, 251)	137 (119, 158)				↓	**
10^−3^·3β,5α,20α-PD, C/U	1.48 (1.22, 1.81)	2.38 (1.87, 3.09)	2.59 (2.27, 2.97)	2.23 (1.97, 2.53)			↑		*
3β,5β,20α-PD, C/U	93.2 (60, 139)	229 (150, 339)	196 (151, 252)	88.9 (65.9, 118)			↑	↓	**
11β-OH-3α,5α-THA, C/U	13.4 (11.6, 15.5)	16.9 (14.4, 19.8)	22 (20.2, 24)	18.9 (17.4, 20.5)					*
11β-OH-3β,5α-THA/3β,5α-THA	0.376 (0.309, 0.453)	0.322 (0.262, 0.391)	0.193 (0.17, 0.217)	0.282 (0.253, 0.313)				↑	*
10^3^·16α-OH-PREG/PREG	324 (293, 359)	371 (333, 414)	502 (474, 533)	399 (377, 423)				↓	**
10^3^·(11β-OH-3α/β,5α-THA)/11β-OH-A	89.6 (78.3, 102)	76 (65.1, 88)	53.2 (48.5, 58.3)	71.9 (66.3, 77.9)				↑	**
(5β,20α-THP+3α/β,5β,20α-PD)/20α-DHP, C	16.6 (14.2, 19.4)	23.9 (20.2, 28.3)	17.1 (15.7, 18.6)	15.7 (14.5, 17.1)			↑		*
(5β,20α-THP+3α/β,5β,20α-PD)/20α-DHP, C+U	13.2 (11.4, 15.3)	19.8 (16.7, 23.5)	17.9 (16.1, 20)	14.9 (13.4, 16.6)			↑		**
3α,5α,20α-PT/3β,5α,20α-PT	1.13 (0.976, 1.3)	0.976 (0.834, 1.14)	0.965 (0.892, 1.04)	1.26 (1.17, 1.36)				↑	*
3α,5α-THA/3β,5α-THA, C	4.03 (3.7, 4.38)	4.6 (4.21, 5)	4.98 (4.75, 5.2)	4.56 (4.36, 4.77)					*
3α,5β-THA/3β,5β-THA, C	3.34 (2.91, 3.82)	2.86 (2.45, 3.32)	3.23 (2.99, 3.5)	3.99 (3.71, 4.29)				↑	*
3α,5β,20α-PD/5β,20α-THP, C	56.5 (48.9, 65.4)	86.9 (74.3, 102)	75.4 (69.3, 82.2)	77.4 (71.3, 84)	↑	*	↑		*
10^3^·3α,5α,17β-AD/3α,5α-THA	63.2 (57.9, 69)	60.5 (55, 66.8)	45.1 (43.1, 47.3)	54.3 (51.8, 57)				↑	*

The abbreviations are the same as in Table 1, Table 2 and Table 3.

## Data Availability

The original contributions presented in the study are included in the article/Supplementary Materials, further inquiries can be directed to the corresponding author/s.

## Supplementary Materials

The following supporting information can be downloaded at: https://www.mdpi.com/article/10.3390/ijms25168729/s1.

## Abbreviations

5α-DHA	5α-Androstane-3,17-dione
5α-DHT	5α-Dihydrotestosterone
3α,5α-THA	Androsterone
3β,5α-THA	Epiandrosterone
3α,5β-THA	Etiocholanolone
3α,5β-THA	Epietiocholanolone
5-HT_3_	Type-3 5-Hydroxytryptamine receptor (serotonin receptor)
8-iso-PGF2α	8-iso-Prostaglandin F_2α_
σ1R	σ1-Receptor
A	Androstenedione
AD	Androstanediol
ADIOL	Androstenediol
AKR1C1	Aldo-keto reductase family 1 member C1
AKR1C2	Aldo-keto reductase family 1 member C2
AKR1C3	Aldo-keto reductase family 1 member C3
AKR1D1	5β-Reductase
ALLO	3α,5α-Tetrahydroprogesterone, allopregnanolone
AMPA	α-Amino-3-hydroxy-5-methyl-4-isoxazolepropionic acid
AMPAR	AMPA receptor
ANOVA	Analysis of variance
AT	Androstenetriol (5-androstene)
CI	Confidence interval
C	Conjugated steroid
C+U	Conjugated + unconjugated steroids
CLCN3	Chloride voltage-gated channel 3
CNS	Central nervous system
CYP11A1	Cholesterol desmolase
CYP11B1	11β-Hydroxylase
CYP17A1	C17-hydroxylase-C17,20-lyase
CYP19A1	Aromatase
CYP21A2	21-Hydroxylase
CYP3A4	Cytochrome P450 3A4
CYP3A7	Cytochrome P450 3A7
CYP7B1	7α-hydroxylase
HSD3B1	Type 1 3β-Hydroxysteroid dehydrogenase
HSD3B2	Type 2 3β-Hydroxysteroid dehydrogenase
HSD3Bs	Type 1 and 2 3β-Hydroxysteroid dehydrogenases
HSD11B1	Type 1 11β-Hydroxysteroid dehydrogenase
HSD11B2	Type 2 11β-Hydroxysteroid dehydrogenase
HSD17B2	Type 2 17β-Hydroxysteroid dehydrogenase
HSD17B3	Type 3 17β-Hydroxysteroid dehydrogenase
HSD17B6	Type 6 17β-Hydroxysteroid dehydrogenase (3α/β epimerase)
DHEA	Dehydroepiandrosterone
DHEAS	Dehydroepiandrosterone sulfate
DHP	Dihydroprogesterone
FEP	First-episode patient
GABA	γ-Aminobutyric acid
GABA_A_R	Type A γ-aminobutyric acid receptor
GC-MS/MS	Gas chromatography tandem mass spectrometry
HC	Healthy control
HPA	Hypothalamic-pituitary adrenal
IL-6	Interleukin 6
IL-17	Interleukin 17
L-type VGCC	L-type voltage gated calcium channels
LLR	Logarithm of likelihood ratio
LSD	Least significant difference
MC	LSD multiple comparisons
mRNA	Messenger ribonucleic acid
NAS	Neuroactive steroid
NMDAR	N-methyl-D-aspartate receptor
OPLS	Orthogonal predictions to latent structure
P	Progesterone
PD	Pregnanediol
PGF_2α_	Prostaglandin F_2α_
PNS	Peripheral nervous system
PREG	Pregnenolone
PREGS	Pregnenolone sulfate
PT	Pregnanetriol
ROS	Reactive oxygen species
SRD5A1	Type 1 5α-reductase
SRD5A2	Type 2 5α-reductase
SULT2A1	Steroid sulfotransferase type 2A1
STS	Steroid sulfatase
T	Testosterone
THP	Tetrahydroprogesterone
TNFα	Tumor necrosis factor α
TRPC5	Short transient receptor potential channels 5
TRPV1	Vanilloid receptor
TRPM3	Melastatin receptor
